# Understanding Fc function for rational vaccine design against pathogens

**DOI:** 10.1128/mbio.03036-23

**Published:** 2023-12-19

**Authors:** Kathryn A. Bowman, Paulina Kaplonek, Ryan P. McNamara

**Affiliations:** 1 Ragon Institute of MGH, MIT, and Harvard, Cambridge, Massachusetts, USA; 2 Division of Infectious Diseases, Brigham and Women’s Hospital, Boston, Massachusetts, USA; Ohio State University, Columbus, Ohio, USA

**Keywords:** antibody, Fc receptor, Fc-effector function, vaccine, vaccine design

## Abstract

Antibodies represent the primary correlate of immunity following most clinically approved vaccines. However, their mechanisms of action vary from pathogen to pathogen, ranging from neutralization, to opsonophagocytosis, to cytotoxicity. Antibody functions are regulated both by antigen specificity (Fab domain) and by the interaction of their Fc domain with distinct types of Fc receptors (FcRs) present in immune cells. Increasing evidence highlights the critical nature of Fc:FcR interactions in controlling pathogen spread and limiting the disease state. Moreover, variation in Fc-receptor engagement during the course of infection has been demonstrated across a range of pathogens, and this can be further influenced by prior exposure(s)/immunizations, age, pregnancy, and underlying health conditions. Fc:FcR functional variation occurs at the level of antibody isotype and subclass selection as well as post-translational modification of antibodies that shape Fc:FcR-interactions. These factors collectively support a model whereby the immune system actively harnesses and directs Fc:FcR interactions to fight disease. By defining the precise humoral mechanisms that control infections, as well as understanding how these functions can be actively tuned, it may be possible to open new paths for improving existing or novel vaccines.

## INTRODUCTION

Vaccine campaigns to date have resulted in a significant reduction of morbidity and mortality across the ages, estimated to prevent approximately 2–3 million deaths annually (1, 2). However, new vaccines are still urgently needed to combat widely circulating agents such as *Mycobacterium tuberculosis*, human immunodeficiency virus (HIV), *Plasmodium*, SARS-related coronaviruses, etc. In addition, new vaccination strategies are needed to address disease prevalence in vulnerable populations that respond poorly to current vaccine strategies such as the elderly, neonates, immunocompromised, etc. Thus, while existing vaccine platforms have successfully revolutionized the battle against many pathogens to date, a deeper appreciation of immune mechanisms of protection is needed for next-generation formulations and platforms.

Induction of antibodies represents the primary correlate of protection against infection/disease for most vaccines (3). Emerging data clearly illustrate that vaccines drive protection *via* mechanistically distinct antibody functions including (i) neutralization, (ii) opsonophagocytosis, (iii) cytokine secretion, (iv) inflammatory cell degranulation, and (v) complement deposition and several others. For example, neutralizing antibodies are crucial for protection against measles (4, 5), opsonophagocytic antibodies are key to defense against *Streptococcus pneumoniae* (6), and antibodies able to drive cytotoxic destruction of infected cells are linked to immunity against influenza (7, 8). It is important to note that these functions frequently synergize with each other to confer protection, and analysis of one function alone is likely not sufficient to account for the entirety of “protective immunity.” Recently developed sophisticated functional immune assays that investigate antibody mechanism of action represent a more pivotal readout of immunity and marker of true clinical efficacy. This review aims to review antibody mechanisms of protection across pathogens, explore the mechanisms by which these antibody functions are driven selectively by the immune system, and discuss novel strategies to harness these functions for future vaccine development.

## MECHANISMS OF ANTIBODY-MEDIATED PROTECTION

**Fig 1:**
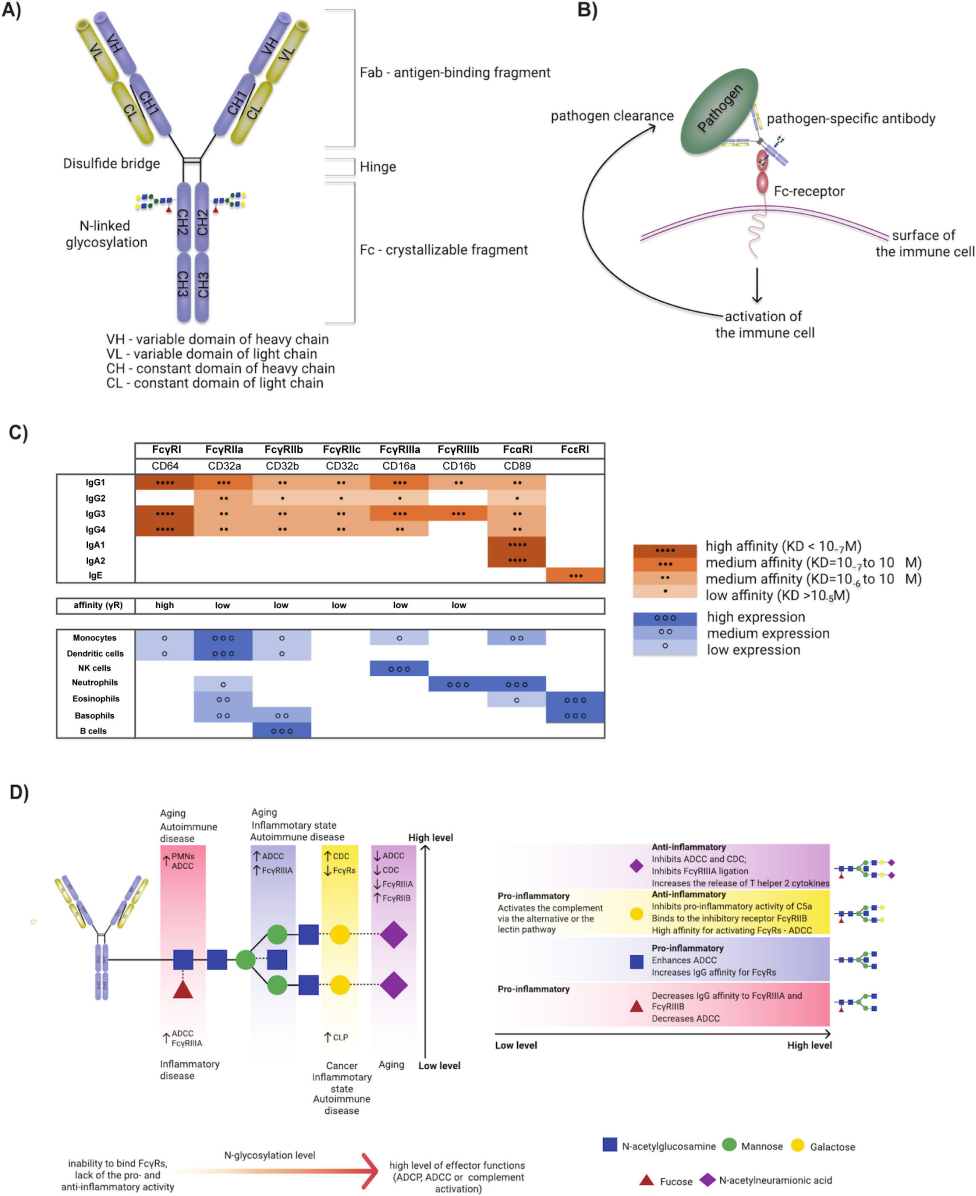
IgG structure and Fc-mediated effector mechanisms and antibody glycosylation. (**A**) An IgG consists of two fragments, an antigen-binding fragment (Fab) and a crystallizable fragment (Fc) connected by a flexible hinge region. The Fab region includes variable and constant domains both on the heavy (VH and VL) and light chains (CL and CH1). The Fc fragment contains complementary CH2 and CH3 domains with an N-linked glycan attached to a conserved glycosylation site at Asn297 of the CH2 (simplified picture). (**B**) Antibodies recognize and bind to pathogens *via* the Fab fragment. The Fc-region, then, interacts with Fc-receptors (FcRs) present in various types of immune cells. (**C**) Antibody subclasses and isotypes bind to FcRs with different affinities and are differentially expressed across various cell types. (**D**) The structure of the biantennary N-linked glycan, attached to asparagine 297 of the IgG heavy chain constant domain (CH2), sits between the arms of the Fc and regulates antibody function by altering the structure of the Fc. Changes in the shape and flexibility of the Fc alter the affinity of the Fc for Fc receptors. The core glycan comprises of two consecutive *N*-acetylglucosamine (GlcNAc) molecules, followed by a mannose and two additional mannose antennae, each with a single GlcNAc attached. In addition, four sugars can be added in variable combinations. Two galactoses can be added to the antennary GlcNAcs (galactosylation), followed by sialic acid that can be added to each of the galactose residues (sialylation). In addition, a single GlcNAc can be added to the core mannose, creating a bisecting arm (bisection). Finally, fucose can be added to the first GlcNAc (fucosylation).

Antibodies recognize pathogens through their antigen-binding fragments (Fab). Each human immunoglobulin is composed of two Fab domains and a constant crystallizable fragment (Fc fragment) domain. The Fab fragments are composed of a heterodimer of a light chain (LC) and a heavy chain (HC). The Fc-domain is composed of HC only and linked to the Fab *via* a hinge region (Fig. 1A) (9). The Fc domain interacts with effector cells *via* their Fc-receptors (FcR) and/or complement receptors (Fig. 1B). The Fc domain binds to specialized cell-surfaced and secreted receptors, including FcR, complement, and other non-canonical receptors found on immune cells (10). Thus, the Fab provides antigen specificity, tethering the antibody to the pathogen or pathogen-associated peptides, whereas the Fc links the adaptive (Fab) and innate (FcR) immune systems.

The Fc-domain is a multifaceted linker, providing specific instructions to the innate immune system on how it should process the target to which the antibody binds. FcRs recognize the Fc regions of IgG antibodies and include Fc-gamma receptor I (FcγRI, also known as CD64), FcγRII (also known as CD32), and FcγRIII (also known as CD16). These will be referred to as FcγR throughout this review. Additional FcRs exist for other antibody isotypes including FcϵRI:IgE, FcαRI/CD89:IgA, FcμR:IgM, and Fcα/μR:IgA/IgM (11). The wide array of FcRs provides a critical mechanism for antibodies of distinct isotypes to interact with and activate signaling cascades of numerous cells of the immune system upon pathogen recognition.

IgG can also bind to non-canonical receptors such as the neonatal Fc receptor (FcRn). FcRn binds the Fc-domain of IgG with high affinity at low pH, allowing for efficient capture and release of antibodies across cell barriers. This mechanism also plays an essential role in serum IgG homeostasis. In addition to their role in trafficking IgG1, FcRn is expressed on multiple immune cells, such as dendritic cells (DCs), macrophages, monocytes, neutrophils, and B cells, to facilitate the uptake and degradation of IgG-opsonized pathogens. For example, the association of hemaglutinin (HA) head-specific antibody-viral complexes with FcRn prevents viral ribonucleoprotein transport to the nucleus, preventing virus replication (12). In addition to FcRs, complement receptors (CRs) expressed by antigen-presenting cells (APCs) are essential for mediating effector function. Complement proteins C3b and C4b, deposited on immune complexes following C1q engagement, can bind to the transmembrane CR1 found on innate immune cells such as neutrophils, monocytes, and dendritic cells. The binding of the C3b-immune complex to membrane CR1 on antigen-presenting cells induces opsonization and antigen presentation in the context of major histocompatibility complex (MHC) molecules to enhance T-cell immunity (13). In addition, C3b, as well as other derivatives iC3b, C3dg, and C3d, bind to the B-cell receptors CR2 and CR1 and play a key role in enhancing B-cell immunity by increasing their entrance and survival in germinal centers (GCs) as well as stimulating memory B-cell development (14, 15).

FcRs are expressed in diverse combinations on different cell types, which leads to unique response patterns. The majority of innate effector cells (monocytes, macrophages, DCs, basophils, and mast cells) co-express both activating FcRs (FcγRI, FcγRIIa, and FcγRIIIa) and inhibitory FcR (FcγRIIb). This allows for the balancing of Ig-mediated cellular activation (16). FcγRI binds to IgG much more potently than other receptors. FcγRII and FcγRIII are characterized by a low affinity for IgG (with K_D_ for human IgG1 = 10^−5^–10^−7^M) and are also able to engage multimeric immune complexes (IgG-antigen complexes) through high avidity interactions (17).

Antibody isotype/subclass selection and glycosylation collectively shape the binding affinity of antibodies to FcRs and complement. Following initial antigen recognition, either infection or immunization, affinity maturation of the Fab and class switch recombination (CSR) of the Fc lead to the production of higher affinity IgGs of different subclass/isotypes (18, 19). The process leads to the directed combination of particular antigen specificities with unique functions *via* the maintenance or replacement of the Ig heavy chain (HC) with one of 10 distinct constant regions [e.g., Cμ (IgM), Cγ (IgG1-4), Cα (IgA1 or 2), or Cε (IgE)] each with a unique affinity for Fc-receptors and complement (20). Humans possess four IgG subclasses (IgG1, IgG2, IgG3, and IgG4) with different functionality and affinity for FcγRs (21). IgG subclasses demonstrate different affinities for FcγRs, with IgG3 having the highest affinity for most FcγRs, followed by IgG1, IgG4, and IgG2 (Fig. 1C) (22).

In addition to isotype and subclass selection, changes in Fc-glycosylation at conserved sites on the antibody-heavy chain profoundly alter antibody interactions with FcRs (Fig. 1D). For example, when the N-glycan is enzymatically cleaved from IgG, the antibody’s ability to bind to FcγRs is abrogated (23). Given the importance of IgG Fc-glycosylation on shaping FcγR binding, specific Fc-glycan structures have been linked to distinct Fc-effector functions. Along these lines, a loss of fucosylation (afucosylation) improves IgG affinity for FcγRIIIa (24). In addition, increased sialylation has been linked to anti-inflammatory functions (25).

Shifts in glycosylation patterns have been documented in different pathological and physiological conditions such as between autoimmune and infectious diseases, as well as age, vaccination, and pregnancy. Specifically, reproducible shifts toward enhanced agalactosylated antibodies emerge under chronic inflammatory conditions and a rapid loss of galactose on disease-specific antibodies precedes the onset of autoimmune flares (26). After vaccination, antigen-specific antibody populations are decorated with different Fc-glycans (27, 28). Moreover, these glycans shift in a pathogen-specific manner (29). Similarly, glycan signatures can shift depending on a vaccine adjuvant, even if the vaccine is targeted to the same antigen (29). These data collectively suggest that antibody glycosylation is a highly regulated process (30). Intracellularly, a complex cascade of glycosyltransferases and glycosidases acts to shape the Fc-glycan in a sequential and highly organized manner in the Golgi apparatus. Analysis of the sialyltransferase St6Gal1 and the fucosyltransferase Fut8 expression in plasmablasts and memory B cells at different time points following trivalent influenza virus vaccination has demonstrated significant shifts in enzyme expression, likely to be key to shaping the antibody glycans (31). Along these lines, alterations in Fc glycoforms on HA-specific IgGs have been directly linked to glycosyltransferase expression profiles (30).

The Fab represents an evolutionary marvel, permitted to evolve genetically, *via* somatic hypermutation, to explore a nearly infinite number of sequence-based solutions to develop ultra-specific binding to an antigen. This process of evolution and integrated perpetual selection within the germinal center (GC) of the lymph node enables the selection of antibodies to mount a multipronged targeting program to a pathogen at distinct regions of interest. The ability of B cells to create this diversified repertoire of binding antibodies is owed to their somatic hypermutation (SHM) through mechanisms such as activation-induced cytidine deaminase (AID). Cytidine deamination converts the nucleotide to uracil, which can result in (1) C → T mutation during replication as uracil will be read as thymine by DNA polymerase; (2) activation of the double-strand break repair pathway and translesion DNA synthesis, resulting in mutations at the C or neighboring nucleotides; and (3) non-canonical mismatch repair pathway activation in which the highly mutagenic DNA polymerase η is recruited to the G:U mismatch (32–35). This entire mutagenic process occurs largely within the variable (*v*) and diversity (*d*) gene segments of the immunoglobulin loci within B cells (36–38). The hotspots of these mutations within the *v* and *d* gene segments correspond to the complementarity-determining regions (CDR). These CDRs, located on the heavy chain of the immunoglobulin, are responsible for the direct contact of the Fab with the antigen (39–41). Therefore this deliberate, gene-segment localized mutagenic process can give rise to a bevy of unique antibody-producing B cells. Immunoglobulins produced by B cells that exhibit low affinity to the target antigen undergo apoptosis whereas B cells producing antibodies of strong antigen binding potential are expanded (42). *Via* this process, the immune system is progressively populated by waves of antibodies that evolve to bind more rapidly, more specifically, and with higher affinity to target antigens of interest with a diversity of functions.

A notable class of antibodies are those that specifically recognize regions of pathogens used to mediate penetrance. These “neutralizing antibodies” selectively displace or interfere with pathogen-antigen binding with stunning precision. For example, neutralizing antibodies to HIV and Dengue virus must bind to specific minimal, often quaternary epitopes, on virion glycoproteins to drive neutralization (43, 44). Similarly, antibodies able to block and displace bacterial toxins, such as those produced by *Corynebacterium diphtheriae* and *Bacillus anthracis*, are also key to vaccine-mediated protection (45, 46). Increasing evidence, however, has illustrated that blocking a toxin or pathogen from accessing entry receptors cannot completely ablate disease. Data increasingly point to the important role of Fc-mediated recruitment of innate immune effector functions in the clearance of toxins and pathogens. For some neutralizing antibodies against viruses, such as HIV, Fc-mediated functions are also essential for protection *in vivo*. In this case, protective activity can be lost if antibody-mediated interactions with FcRs are abrogated (47). Therefore, there exists an intimate collaboration between the Fab and the Fc for protection.

While neutralization is critical for protection against several infections, neutralization is not necessary against all infections (48, 49). For example, in the case of influenza, some antibodies to the stem of the hemagglutinin (HA) antigen, involved in binding and infection, do not neutralize the virus *in vitro*. However, these antibodies can block infection *in vivo* (50, 51). Interestingly, the protective activity of these antibodies depends on the ability of the antibodies to interact with FcRs and appears to depend on the selective recruitment of NK cells to exert antibody-dependent cellular cytotoxicity (ADCC) (52). Moreover, population-level studies have also demonstrated increased levels of antibody-dependent complement deposition (ADCD), and antibody-dependent cellular phagocytosis (ADCP) by myeloid-lineage cells and neutrophils in cohorts of individuals that exhibit a reduced likelihood of infection (49, 53).

### Antibody-dependent cellular phagocytosis

**Fig 2:**
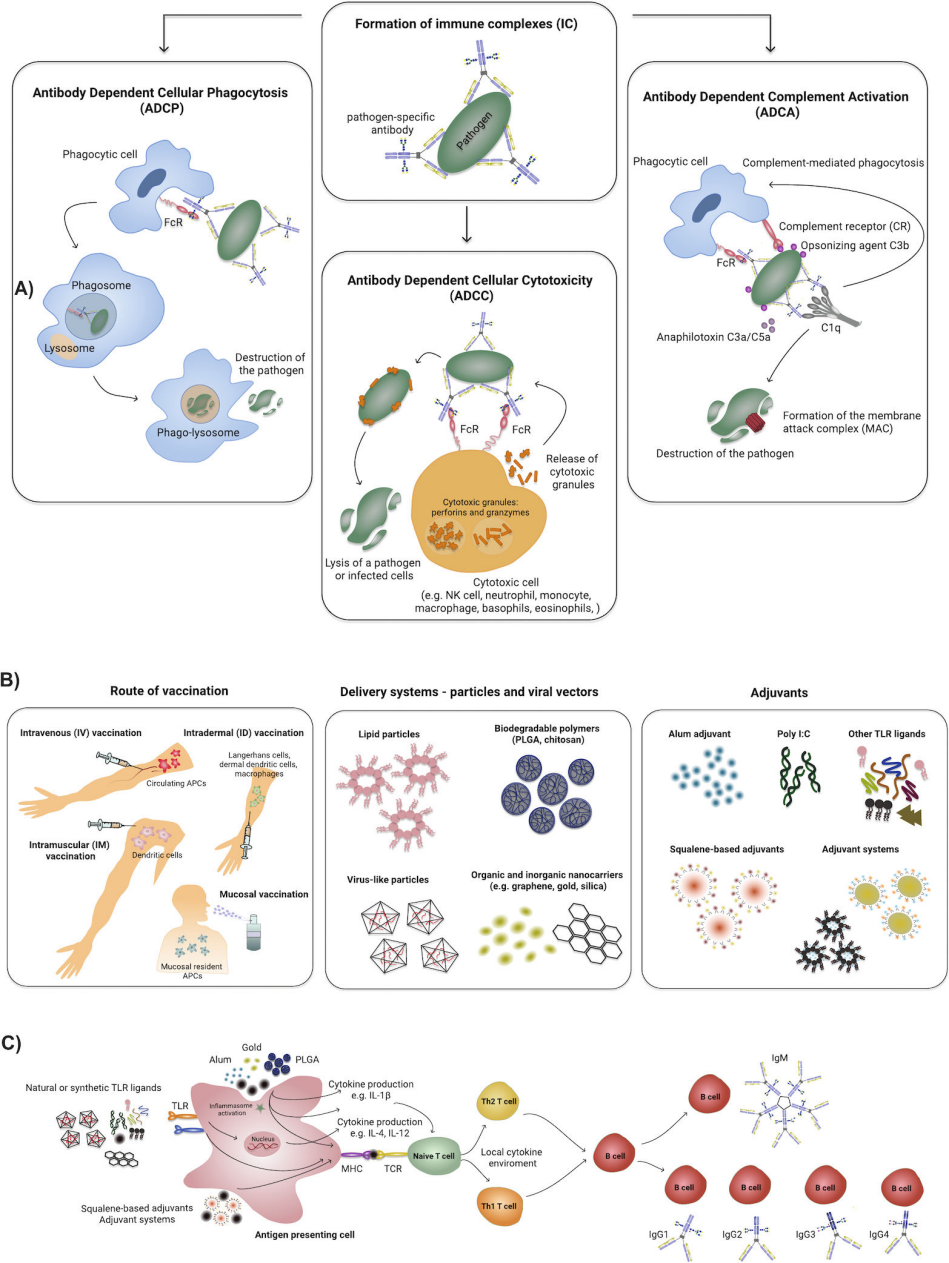
The impact of different routes of vaccination and formulation on Fc-mediated antibody effector functions. (**A**) ADCP, ADCC, and antibody-dependent complement activation are the main Fc-receptor-mediated antibody effector functions leading to the destruction and clearance of the pathogen and therefore protection against infection/disease. Binding of the pathogen-specific antibodies to the surface of the pathogen leads to the formation of immune complexes. The interaction of the Fc-domain of antibodies from immune complexes and Fc-receptors present on multiple types of innate immune cells mediates the downstream effector functions. (**B**) Different routes of immunization, such as intravenous (IV), intradermal (ID), intramuscular (IM), and mucosal, enable to target distinct types of APCs leading to the production of particular cytokines further shaping the antibody type and their Fc-effector functions as well as proper choice of a delivery system and adjuvant initiate distinct early immune response at the site of vaccine administration and therefore influence the Fc-subclass/Fc-glycosylation selection (**C**).

ADCP is the humoral immune function whereby antibody-opsonized pathogens or infected cells bind to FcRs or complement on the surface of phagocytes to drive immune complex uptake (Fig. 2A). Professional phagocytes include monocytes, macrophages, neutrophils, DCs, or eosinophils, all of which express different combinations of FcRs, including FcRs for all antibody isotypes. However, FcR expression varies on these cells in a tissue-dependent manner, where mucosal macrophages and DCs express high levels of FcαRI (CD89), not found on peripheral macrophages and DCs, enabling these cells to respond to antibody-opsonized material in a tissue-specific manner.

The early steps of ADCP depend on the number and combination of FcRs engaged (relative to the ligand and antibody density on the target antigen). The binding of activating classical FcRs leads to the phosphorylation of immunoreceptor tyrosine-based activation motifs (ITAM) (on activating FcRs) and initiation of signaling cascades (54). Given the higher affinity of IgG1 and IgG3 for FcγR binding, immune complexes composed of these antibodies lead to rapid triggering and robust phagocytic clearance of these antibody complexes. In addition, IgA and IgM have also been implicated in rapid and robust immune complex uptake, triggered by both isotype-specific Fc-receptors and complement receptors.

Depending on the immune cell triggered, different inflammatory responses may evolve following phagocytosis. In human blood, neutrophils are a critical first line of defense against pathogens. Due to their unique FcR repertoire, including FcγRIIa (CD32a), FcγRIIIb (CD16b), FcγRI (CD64), and FcαRI (CD89), these innate immune cells represent highly specialized gatekeepers able to rapidly respond to antibody-complexes, drive rapid degradation and clearance, and support antigen cross-presentation (55). However, depending on the FcRs triggered, distinct inflammatory consequences can evolve. For example, IgA/FcαRI binding can lead to rapid neutrophil extracellular trap formation, or NETosis killing (56). Conversely, IgG activation of neutrophils can lead to degranulation and cytokine secretion. DCs, on the other hand, pick up antibody complexes, and depending on the antibody/FcR binding profiles, can lead to lysosomal proteolytic degradation and/or antigen presentation through MHCI and MHCII. This simultaneously allows for a response to existing pathogens and a priming of new memory responses (57). This DC switch is regulated by antibody-binding to the neonatal Fc-receptor, FcRn, which drives uptake in a pH-dependent manner.

Beyond FcRs, immune complex uptake may also occur following complement deposition on immune complexes. In addition to their role in creating pores in membranes, the complement components deposited on immune complexes interact with complement receptors (CR) found in phagocytic immune cells. For example, the first component of the classical complement cascade, C1q, interacts with the C1q-receptor (C1qr) found in myeloid cells (58). In addition, following the activation of C3 and deposition of C3b, iC3b (after cleavage of C3b), and C3d on immune complexes, complement receptors (CRs) can mediate phagocytosis. These receptors include CRIg, the multifunctional CR1, CR2, and the β2-integrin members CR3 and CR4. Surface expression of these complement receptors differs, with CR1 and CR2 being expressed on myeloid, neutrophils B cells, and macrophages, CR3 and CR4 being present on myeloid, neutrophils, activated lymphocytes, and natural killer cells, and CRIg being present on tissue-specific macrophages (13, 28, 59–68). Complement-mediated phagocytosis is particularly essential during the acute immune response to infection, where low-affinity IgMs efficiently recruit C1q and drive rapid and effective phagocytic clearance. In addition, this IgM/C1q phagocytic axis also plays a critical response in protection against T-cell-independent antigens. Moreover, because T cells also express C3aR, C5aR1, and C5aR2, adaptive immune cells may also be activated by antibody complexes, resulting in broader immune activation (69–72). Given the extensive expression of CRs, these receptors likely collaborate with FcRs to drive clearance, intracellular routing, and immune activation/signaling to nearby cells.

ADCP has been implicated in the protection against bacteria (73), parasites (61), fungi (74), and viruses (75). Opsonophagocytosis plays an important protective role against *Streptococcus pneumoniae*, *Staphylococcus aureus*, *Pseudomonas aeruginosa,* and *Klebsiella pneumoniae*. Surface-exposed proteins and cell wall components are targets for antibodies able to interact with FcγRs and CRs, leading to rapid bacterial uptake and destruction. For example, meningococcal-specific polysaccharide-specific IgG2 that interacts with FcγRIIa leads to increased killing *via* neutrophils (73, 76). Similarly, neutrophil-mediated opsonophagocytic killing plays a critical role in immunity against fungal *Candida albicans* infection (77), shown recently to be critical in the defense against multidrug-resistant fungal pathogens (74).

Recent evidence points to a critical role for opsonophagocytosis in antiviral and anti-parasitic immunity. For example, vaccine-induced antibody-mediated phagocytosis has been linked to protection against the simian immunodeficiency virus (SIV), the evolutionary precursor of HIV (78). Interestingly, using the same DNA/Adenoviral-based SIV vaccine, monocyte recruiting IgG phagocytic responses were induced by intra-muscular vaccination, whereas IgA-driven neutrophil phagocytic responses were induced by intranasal immunization, both resulting in ~50% protection from infection. Given the presence of higher levels of phagocytic antibodies among human spontaneous controllers of HIV infection (79) and our emerging appreciation for the role of homozygosity for the low-affinity FcγRIIa allele among HIV rapid progressors (defined by a low CD4^+^ T-cell count) (80), these data highlight the role of opsinophagocytosis in HIV control. Moreover, the data point to opportunities to drive distinct phagocytic responses, *via* monocytes or neutrophils, by vaccination route.

Phagocytic responses by neutrophils were linked with long-term vaccine-mediated protection against Ebolavirus (EBOV) in non-human primates (81). Interestingly, phagocytic responses correlated with survival post-virus challenge were against the truncated EBOV glycoprotein (GP), termed the soluble GP (or sGP) (81–83). These functionally leveraged antibodies were more clear correlates of protection than neutralizing antibodies themselves. The vaccine platform was a live-attenuated vesicular stomatitis virus that encodes for EBOV GP, administered intramuscularly (84). This again hints at vaccination platform-based and/or immunization sites as key determinants of antibody functions (see section Prospects for Fc programming).

The COVID-19 pandemic provided unprecedented insight into how ADCP functions as a novel pathogen. Humoral immunity was low in the human population prior to 2019, allowing for the generation of a novel, polyfunctional antibody response through vaccination and/or recovery. The primary target of SARS-CoV-2-directed antibodies is the spike glycoprotein. ADCP of the spike is mediated through antibodies recognizing the viral protein at multiple subdomains (85–90), whereas neutralizing antibodies are much more specifically targeted to the receptor binding domain. The presence of ADCP-leveraged antibodies is linked to positive clinical outcomes and protection against antigenically drifted SARS-CoV-2 variants (85, 86, 91).

Similarly, vaccine-induced phagocytic antibodies against malaria have been implicated in the protection against parasitic infection (92). Recent immune correlates analysis of the most advanced malaria vaccine candidate, RTS’S (93, 94), have pointed to a critical role for phagocytosis as a key mechanism of protection following vaccination (92). While decades of data had clearly linked protection from infection following vaccination to vaccine-induced titers, functional characterization of these antibodies pointed to an enrichment of phagocytic functions in individuals able to resist the *Plasmodium* challenge (93).

Given the abundance of phagocytic cells in the skin, blood, and liver, it remains unclear where this protective activity may occur on a pathogen-by-pathogen basis. For example, the presence of spike-reactive IgG1 in the lungs was a correlate of protection for COVID-19 but compartment-specific functions of ADCP have not yet been delineated for COVID-19. This is also true for *Plasmodium*, which has a multi-compartment life cycle. Future studies into how ADCP is regionally activated are warranted.

### Antibody-dependent cellular cytotoxicity

ADCC represents the mechanism by which innate effector cells (lacking specificity for an antigen) recognize antibody-opsonized pathogens/antigens, or entire infected cells, and trigger the cytotoxic destruction of that antibody-opsonized target (Fig. 2A). Cytotoxicity is driven by the release of cytotoxic granules containing perforins and granzymes from cytotoxic effector cells. ADCC is best described for IgG-mediated natural killer cell (NK) activation. Specifically, IgG1 and IgG3 appear to drive ADCC most effectively due to their affinity for FcγRIIIa (CD16), a dominant FcγR found in NK cells. However, the engagement of FcγRI (CD64) and FcγRII (CD32) has also been implicated in macrophage-, neutrophil-, or eosinophil-mediated degranulation and cytotoxicity (16). Furthermore, emerging data point to a role for IgA-FcαR and IgE-FcεR as additional mediators of ADCC by neutrophils, particularly in the setting of cancer treatments (95, 96). ADCC-like activities can also occur in cytolytic granule-producing neutrophils, monocytes, macrophages, basophils, eosinophils, NK T cells, αβ, and γδ CD8+ T cells, *via* Fc-mediated activation (97). The distinct granule contents found in each of these cell types can result in a remarkable breadth of distinct inflammatory and cytotoxic outcomes.

ADCC has been implicated in protection against multiple viruses and some intracellular bacteria and parasites. ADCC can act both indirectly to eliminate the pathogen within infected cells as well as directly on particular pathogens. For example, ADCC activity has been linked to disease control following HIV, influenza virus, Ebolavirus, or respiratory syncytial virus (RSV) challenge(s). Several studies have noted enhanced ADCC-inducing antibodies among HIV spontaneous controllers compared to individuals progressing to AIDS (98). Similarly, both animal and human studies have demonstrated a critical role for ADCC in the control of influenza, where survival and recovery following H7N9 infection were preferentially observed in the presence of anti-HA antibodies able to activate primary NK cells (99). Conversely, non-survivors of both HIV and influenza infections generate lower levels of FcγR binding antibodies and reduced ADCC (99). Along the same lines, reduced NK cell counts have been observed in hospitalized children with RSV lower respiratory infection (100). Moreover, NK cell-activating monoclonal antibodies have been shown to provide enhanced protection against Ebolavirus, particularly for antibodies targeting the base of GP (101). For COVID-19, the kinetics of FcγRIIIa-binding antibodies were linked to severe disease survival (91). Finally, as mentioned above, cross-influenza protective antibodies that target the HA-stem, which is more conserved across influenza viruses, utilize ADCC-like activities for protection (102). Collectively, the ability to recruit NK cells appears key to protection against a variety of viruses.

Interestingly, ADCC has also been implicated in protection against intracellular bacteria as well as some parasites. Because cytotoxic granules must fuse with the target membrane and release granzymes into the cell to drive destruction, canonical ADCC activity is not compatible with all pathogen membranes. For example, NK cells may have a limited capacity to eliminate highly coated bacteria, but emerging data point to a critical role for NK cells in the destruction of intracellular bacteria once inside monocytes/macrophages. Specifically, both elevated levels of FcR-expressing NK cells (103) and enhanced ADCC-inducing antibodies have been observed in controlled *Mycobacterium tuberculosis* (Mtb) infection compared to individuals with active, uncontrolled disease (104). Likewise, NK cells have been implicated in the control of parasite infections such as malaria and trypanosomiasis. For *Plasmodium*, NK cells have been implicated in killing the parasite during the blood stage by attacking infected red blood cells (105) and eliminating the liver stage of the infection *via* the killing of infected hepatocytes (106). Similarly, ADCC has been implicated in the direct elimination of *Trypanosoma cruzi* (107), mechanistically pointing to FcRγIIIa-dependent ADCC activity as a key mechanism for the direct elimination of these pathogens.

### Antibody-dependent complement activation

In addition to the directed role of innate immune cells, antibodies can also recruit the complement cascade to destroy pathogens. Complement is a complex of soluble blood proteins that can be deposited to mark antibody complexes for destruction. Complement is an evolutionarily conserved process that represents one of the first lines of innate immune defense against many bacteria, yeasts, and viruses. Activation of the complement cascade can lead to direct or indirect destruction of pathogens through (i) the formation of a self-organized membrane attack complex (MAC) on the surface of the pathogen membrane, (ii) directed pathogen uptake, or (iii) the activation of the innate immune system by inflammatory mediators (Fig. 2A).

IgM, followed by IgG3 and IgG1, display the highest potential to activate the classical complement pathway. This pathway is triggered by IgM deposition on a target antigen or the formation of IgG hexamers, either of which allows C1q to bind (63). This initial binding of C1q to surface-bound IgM or IgG results in the autoactivation of C1r and cleavage of C1s. Active C1s cleave C4 and C2, which collectively form the C3 convertase, C4bC2a. The C3 convertase cleaves C3, the major complement protein in serum. Upon cleavage, C3a (an anaphylatoxin) is released, and C3b (the opsonizing agent and ligand for complement receptors) deposits on the surface of the antibody target (14, 108). Complement activation can drive phagocytic uptake or can continue to catalyze the deposition of complement components, leading to the formation of the MAC. The formation of the MAC is blocked on the surface of most human cells by MAC-disassembling mechanisms aimed at preventing non-specific cellular destruction (108). Most pathogens lack these factors, providing an opportunity for the complement to act in pathogen destruction. Interestingly, many enveloped viruses, including HIV, human cytomegalovirus, hepatitis C, and vaccinia virus (62, 109, 110), steal human complement decay activating factor (DAF/CD55) on their viral membranes, allowing them to subvert complement-mediated destruction. However, because C1q aggregates antibodies, complement can enhance viral neutralization by blocking and clustering viral receptors (111) as well as direct viruses to opsonophagocytic uptake.

Complement is essential in the control of multiple bacterial infections, as observed in individuals with complement deficiencies, who experience higher rates of recurrent encapsulated bacterial infections such as *Streptococcus pneumoniae*, *Haemophilus influenzae*, and *Neisseria meningitidis*, especially in early childhood (112). Moreover, complement is key to protection against enteric pathogens including *Salmonella typhimurium* and *Shigella* (113) as individuals with high levels of SBA-inducing antibodies exhibited reduced disease.

In the context of viral pathogens, a study conducted in complement-deficient mice (a classical pathway C1q^−/−^, a lectin pathway C4^−/−^, an alternative pathway factor D (fD)^−/−^, and factor B (fB)^−/−^) demonstrate that all complement activation pathways are essential for control of severe West Nile Virus disease (114). Similarly, complement-mediated viral lysis was observed against HIV in patients with acute HIV infection (115). Complement also plays a key role in vaccinia virus (VACV) neutralization, highlighting the collaboration between the Fab and the Fc in antibody-mediated protection against VACV (protection confirmed in mouse models of VACV infection) (116). Thus, both direct complement-mediated killing and indirect complement-mediated phagocytic clearance of pathogens are key to protection against pathogens.

## PROSPECTS FOR FC PROGRAMMING

Given our mounting appreciation for the role of diverse humoral immune functions in the control and elimination of multiple pathogens, defining novel mechanisms to control and tune both Fab-evolution and Fc-effector function may offer novel opportunities to strategically design more effective vaccines. Distinct delivery platforms and adjuvants have been shown to elicit unique Fc-effector function signatures, even when directed toward the same antigen (117–119). Several approaches exist to tune early inflammatory cues to shape and direct the vaccine-induced humoral immune response (Fig. 2B).

### Route of vaccination

The route of vaccination and the initial types of innate immune cells triggered can dramatically alter the quality of the humoral immune response. Specifically, different innate immune APCs exist within different tissues, programmed to drive tissue-appropriate immunity (120, 121). Given that APCs are loaded with diverse sets of pattern recognition receptors expressed in a tissue-specific manner, APCs can rapidly respond to new antigens. This local and APC-specific activation drives downstream inflammatory responses that shape the adaptive immune response (Fig. 2B). For example, vaccines delivered mucosally target mucosal resident APCs poised to elicit mucosal-centric immunity, including the induction of higher levels of IgA (122). Intramuscular (IM) vaccination results in the recruitment of DCs that drive a systemic-centric immune response, largely populated by IgG. The skin is highly enriched in multiple subsets of professional APCs including Langerhans cells, dermal dendritic cells (dDCs), and macrophages, and thus can respond aggressively to novel antigens (123). Along these lines, multiple studies have posited that intradermal delivery of influenza vaccines results in improved immunogenicity and more durable immune responses than the typical IM route (124–127). Also, intravenous vaccination approaches against malaria (128) and *Mycobacterium tuberculosis* (129) showed strong protection related to the induction of enhanced tissue-resident immunity within the liver and lung, respectively.

The IM route of vaccination can stimulate highly functional IgG responses. This is true for a variety of platforms including mRNA (87, 130, 131), live-attenuated (132), inactivated (133), and component-based vaccines (134). These functions include the FcγR-leveraged functions such as ADCP (both myeloid-lineage and neutrophil), ADNK, and ADCD. Interestingly, stimulation of Fc-effector functions through IM vaccination appears to also be bolstered through localized inflammation induced by adjuvants (134, 135). Intranasal (IN) route of vaccination has also been shown to stimulate a durable humoral response, including antibody effector functions. In a study comparing IM and IN vaccination, ADCP by neutrophils was significantly higher in IN-delivered vaccinations, while ADCP driven by monocytes was similar between IM and IN vaccination groups (136). Thus, the route of delivery can result in unique antibody programming and can elicit more tissue/compartment targeted protection.

### Adjuvants

While the route can shape the localization of the immune response, additional inflammatory signals delivered at the time of immunization have been shown to be key to shaping both the Fab and Fc-of the vaccine-induced immune response. Critically, the co-delivery of particular adjuvants, such as synthetic TLR ligands (e.g., TLR4 ligand—monophosphoryl lipid A (MPLA) and TLR7 ligand—Imiquimod) have been demonstrated to drive enhanced affinity maturation and epitope spreading in influenza vaccines in rhesus macaques (137). However, adjuvants have also been shown to drive distinct cytokine and chemokine expression profiles, shaping both T-helper quality and antibody subclass/isotype selection (Fig. 2B and C). For example, the use of alum, the squalene-based oil-in-water emulsion MF59, and other adjuvants in mice drive enhanced functional IgG2a selection compared to standard largely IgG1 selection (138). Poly I:C elicits a highly balanced IgG1, IgG2b, and IgG2c response compared to alum or Addavax (139), pointing to the potential rational selection of adjuvants that give skewed or directed subclass selection in mice. Moreover, a study in non-human primates immunized with HIV envelope glycoprotein gp140 in different adjuvant formulations demonstrated adjuvant-induced differences in antibody glycan profiles, which further correlated with Fc-mediated effector functions (29). Specifically, formulation of the antigen with alum/TLR7 adjuvant resulted in the production of antibodies that strongly bound to the rhesus FcγRs: Rh.RcγRIIa, Rh.FcγRIIb, and Rh.FcγRIIIa and exhibiting high ADCD and ADCP activity (29). Similarly, humans immunized with H5 Influenza HA adjuvanted with MF59 demonstrated higher levels of phagocytic-linked IgG1 and IgG3 *via* enhanced binding to FcγRIIa, but no enhancement in ADCC linked to poor FcγRIIIa binding (140). The increased IgG3 and phagocytic signal in the absence of ADCC were postulated to occur *via* altered Fc-glycosylation, pointing to skewed adjuvant B cell programming (140).

More recently, protein-based COVID-19 vaccines and boosters (prefusion spike) have been shown to elicit highly cross-reactive IgG when administered with the AS03 adjuvant (141–143). Moreover, the presence of AS03 in the immunization formulation was required for functional antibody responses. Notably, FcγR-binding antibodies were robustly induced when the prefusion spike was administered with AS03 compared to a formulation without AS03; in fact, a lower dose prefusion spike + AS03 elicited higher FcγR-binding than a higher dose prefusion spike without AS03 (1 µg prefusion spike + AS03 vs 3 µg prefusion spike −AS03). This was also true for non-neutralizing functions such as ADCD, ADCP, ADNP, and ADNKA where the lower dose vaccine with AS03 outperformed the higher dose vaccine without AS03. These non-human primates immunized with AS03 formulations showed strong protection against the SARS-CoV-2 challenge, and antibodies generated could be passively transferred and confer protection (135). While dissecting the protection afforded exclusively by Fcγ- and/or Fcα-binding antibodies can be challenging in studies like these, it is clear that non-neutralizing antibodies whose functions are leveraged through their Fc domain play a substantial role in disease mitigation. The presence of adjuvants appears highly correlated with this.

Adjuvants appear to selectively skew antibody effector function both through selective isotype/subclass selection and altered Fc-glycosylation. Defining the overall impact of all clinically approved adjuvants may offer an opportunity to customize vaccine and adjuvant combinations based on the desired target immune profile. In addition, understanding what Fc-effector functions remain poorly programmable *via* the currently available adjuvants may point to novel opportunities for the design of novel classes of adjuvants that add value to vaccine development pipelines.

### Delivery systems

Finally, beyond the location of administration and adjuvant technologies, accumulating data suggest that the size, stoichiometry, shape, and arrangement of vaccine antigens can also shape the quality of the immune response (144–147). While single antigens can be less immunogenic than whole pathogens, diverse carrier particles including flexibly shaped and sized biodegradable nanoparticles [such as polylactic acid (PLA) or chitosan] (148, 149), protein nanoparticles (150, 151), inorganic nanocarriers (e.g., gold, carbon, and silica) (152–155) or liposomes (156), can mimic pathogens by triggering specific combinations of receptors, arming the immune system in a highly effective manner (Fig. 2B). Moreover, it has been demonstrated that the controlled release of antigen from particles promotes affinity maturation through increased exposure and perpetual selection of B cells (157).

Distinct delivery systems have been linked with different Fc-mediated functions. Recent studies on COVID-19 vaccinations have given us previously unrealized details on this. For example, mRNA-based vaccines can elicit strong FcγR-binding antibody levels and non-neutralizing functions (87), which have been correlated with reduced disease severity (91). Importantly, while antigenic drift can decrease neutralizing antibody recognition (158, 159), FcγR-binding antibodies and non-neutralizing functions can persist in mRNA-vaccinated individuals. Interestingly, NK-signaling antibodies generated by mRNA vaccines showed a stronger subdomain preference than an inactivated-vaccine-generated response (130, 133). This could be due to how the antigen is presented, with mRNA-based COVID-19 vaccines encoding for a stabilized, prefusion spike, whereas an inactivated COVID-19 vaccine can contain spike in numerous conformations (160–163).

Adenovirus-based delivery systems have also demonstrated an ability to induce functional antibodies that were mostly ambivalent to genetic drift of the antigen, particularly in regards to complement deposition and NK-signaling (164). In addition, recombinant viral vectors can leverage viral carrier danger signals at the time of vaccination aimed at shaping the humoral immune responses. Viral vectors such as adenovirus-based platforms have been successfully employed for COVID-19 vaccines. However, population-based immunity toward the adenovirus vector itself needs to be taken into account when utilizing these platforms because neutralization/inactivation of the vector will decrease antigen production and processing. While emerging data clearly illustrate the highly distinct Fc-effector profiles leveraged by distinct viral vector strategies (165), the specific antibody profiles elicited by each vaccine vector remain incompletely understood. It is tempting to speculate that, like adjuvants, viral vectors could provide strategic information on how immunity is trained to most effectively drive a desired target immune profile.

Discreet mechanistic insight into how various delivery platforms/modalities shape Fc-effector function remains largely unexplored. While it is clear that platforms, adjuvants, and dose schedule impact how Fc functions are leveraged, future studies detailing how this process occurs are desperately needed, especially as the link between Fc functions and clinical outcomes becomes clearer.

## CONCLUSION

While vaccines have revolutionized our ability to limit many previously highly lethal infectious diseases, as well as recently emerged pathogens, current vaccine design strategies and platforms can clearly be improved. This is of particular importance for pathogens with intrinsically high immune escape potential and those with multi-tissue life cycles. With our growing appreciation for the role of both the Fab and the Fc in mechanistic control against pathogens, next-generation vaccine strategies should leverage both ends of the antibody to provide a comprehensive protection strategy.

The COVID-19 pandemic presented itself at a critical moment in shifting this paradigm. The COVID-19 vaccines were developed, manufactured, and distributed within a stunning timeframe. As the pandemic progressed, genetic drift within the SARS-CoV-2 spike, particularly the receptor binding domain, allowed the virus to evade neutralizing antibodies (158, 159, 166–173). However, protection against disease did not see a corresponding drop, signifying that immune correlates of protection beyond neutralization existed. Fc-mediated functions of antibodies are key determinants of COVID-19 disease severity (85, 86, 91, 174–176). Fully dissecting how various platforms, antigen designs, adjuvants, delivery modalities, and routes of administration affect these is critical. Therefore, to accomplish this goal, next-generation vaccine design will require a holistic understanding of the mechanisms of immune protection, taking into account all immune effector functions and the full potential of the humoral immune response. This will require a commitment to integrate and compare immune profiles across geographic, age, sex, gender, etc. demographics, and identifying signatures that predict outcomes. These commitments will not only help to reduce the disease burden of currently circulating pathogens but also allow for the rapid deployment of highly effective vaccines for emerging infectious diseases.

## References

[1] WHO. 2020. Immunization coverage. Available from: https://wwwwhoint/news-room/fact-sheets/detail/immunization-coverage

[2] WHO. 2009. State of the world’s vaccines and immunization. World Health Organization.

[3] van Erp EA, Luytjes W, Ferwerda G, van Kasteren PB. 2019. Fc-mediated antibody effector functions during respiratory syncytial virus infection and disease. Front Immunol 10. doi:10.3389/fimmu.2019.00548 PMC6438959 30967872

[4] Chen RT, Markowitz LE, Albrecht P, Stewart JA, Mofenson LM, Preblud SR, Orenstein WA. 1990. Measles antibody: reevaluation of protective titers. J Infect Dis 162:1036–1042. doi:10.1093/infdis/162.5.1036 2230231

[5] Haralambieva IH, Kennedy RB, Ovsyannikova IG, Schaid DJ, Poland GA. 2019. Current perspectives in assessing humoral immunity after measles vaccination. Expert Rev Vaccines 18:75–87. doi:10.1080/14760584.2019.1559063 30585753 PMC6413513

[6] Schuerman L, Prymula R, Henckaerts I, Poolman J. 2007. ELISA IgG concentrations and opsonophagocytic activity following pneumococcal protein D conjugate vaccination and relationship to efficacy against acute otitis media. Vaccine 25:1962–1968. doi:10.1016/j.vaccine.2006.12.008 17258357

[7] Memoli MJ, Shaw PA, Han A, Czajkowski L, Reed S, Athota R, Bristol T, Fargis S, Risos K, Powers JH, Davey RT, Taubenberger JK. 2016. Evaluation of antihemagglutinin and antineuraminidase antibodies as correlates of protection in an influenza A/H1N1 virus healthy human challenge model. mBio 7:e00417-16. doi:10.1128/mBio.00417-16 27094330 PMC4959521

[8] Boudreau CM, Alter G. 2019. Extra-neutralizing FcR-mediated antibody functions for a universal influenza vaccine. Front Immunol 10:440. doi:10.3389/fimmu.2019.00440 30949165 PMC6436086

[9] Chiu ML, Goulet DR, Teplyakov A, Gilliland GL. 2019. Antibody structure and function: the basis for engineering therapeutics. Antibodies 8:55. doi:10.3390/antib8040055 31816964 PMC6963682

[10] Bournazos S, Ravetch JV. 2015. Fcγ receptor pathways during active and passive immunization. Immunol Rev 268:88–103. doi:10.1111/imr.12343 26497515 PMC7556827

[11] Ben Mkaddem S, Benhamou M, Monteiro RC. 2019. Understanding Fc receptor involvement in inflammatory diseases: from mechanisms to new therapeutic tools. Front Immunol 10:811. doi:10.3389/fimmu.2019.00811 31057544 PMC6481281

[12] Bai Y, Ye L, Tesar DB, Song H, Zhao D, Björkman PJ, Roopenian DC, Zhu X. 2011. Intracellular neutralization of viral infection in polarized epithelial cells by neonatal Fc receptor (FcRn)-mediated IgG transport. Proc Natl Acad Sci U S A 108:18406–18411. doi:10.1073/pnas.1115348108 22042859 PMC3215070

[13] Oliveira LC, Kretzschmar GC, Dos Santos ACM, Camargo CM, Nisihara RM, Farias TDJ, Franke A, Wittig M, Schmidt E, Busch H, Petzl-Erler ML, Boldt ABW. 2019. Complement receptor 1 (CR1, CD35) polymorphisms and soluble CR1: a proposed anti-inflammatory role to quench the fire of "fogo selvagem" pemphigus foliaceus. Front Immunol 10:2585. doi:10.3389/fimmu.2019.02585 31824479 PMC6883348

[14] Gadjeva M. 2014. The complement system. Overview. Methods Mol Biol 1100:1–9. doi:10.1007/978-1-62703-724-2_1 24218246

[15] Rossbacher J, Shlomchik MJ. 2003. The B cell receptor itself can activate complement to provide the complement receptor 1/2 ligand required to enhance B cell immune responses in vivo. J Exp Med 198:591–602. doi:10.1084/jem.20022042 12925675 PMC2194168

[16] Nimmerjahn F, Ravetch JV. 2008. Fcgamma receptors as regulators of immune responses. Nat Rev Immunol 8:34–47. doi:10.1038/nri2206 18064051

[17] Kang TH, Jung ST. 2019. Boosting therapeutic potency of antibodies by taming Fc domain functions. Exp Mol Med 51:1–9. doi:10.1038/s12276-019-0345-9 PMC6859160 31735912

[18] de Taeye SW, Rispens T, Vidarsson G. 2019. The ligands for human IgG and their effector functions. Antibodies (Basel) 8:30. doi:10.3390/antib8020030 31544836 PMC6640714

[19] Stavnezer Janet, Guikema JEJ, Schrader CE. 2008. Mechanism and regulation of class switch recombination. Annu Rev Immunol 26:261–292. doi:10.1146/annurev.immunol.26.021607.090248 18370922 PMC2707252

[20] Stavnezer J, Schrader CE. 2014. IgH chain class switch recombination: mechanism and regulation. J Immunol 193:5370–5378. doi:10.4049/jimmunol.1401849 25411432 PMC4447316

[21] Irani V, Guy AJ, Andrew D, Beeson JG, Ramsland PA, Richards JS. 2015. Molecular properties of human IgG subclasses and their implications for designing therapeutic monoclonal antibodies against infectious diseases. Mol Immunol 67:171–182. doi:10.1016/j.molimm.2015.03.255 25900877

[22] Bruhns P, Iannascoli B, England P, Mancardi DA, Fernandez N, Jorieux S, Daëron M. 2009. Specificity and affinity of human Fcγ receptors and their polymorphic variants for human IgG subclasses. Blood 113:3716–3725. doi:10.1182/blood-2008-09-179754 19018092

[23] Anthony RM, Nimmerjahn F. 2011. The role of differential IgG glycosylation in the interaction of antibodies with Fcγrs in vivo. Curr Opin Organ Transplant 16:7–14. doi:10.1097/MOT.0b013e328342538f 21150612

[24] Falconer DJ, Subedi GP, Marcella AM, Barb AW. 2018. Antibody fucosylation lowers the FcgammaRIIIa/CD16a affinity by limiting the conformations sampled by the N162-glycan. ACS Chem Biol 13:2179–2189. doi:10.1021/acschembio.8b00342 30016589 PMC6415948

[25] Kaneko Y, Nimmerjahn F, Ravetch JV. 2006. Anti-inflammatory activity of immunoglobulin G resulting from Fc sialylation. Science 313:670–673. doi:10.1126/science.1129594 16888140

[26] Dekkers G, Rispens T, Vidarsson G. 2018. Novel concepts of altered immunoglobulin G galactosylation in autoimmune diseases. Front Immunol 9:553. doi:10.3389/fimmu.2018.00553 29616041 PMC5867308

[27] Selman MHJ, de Jong SE, Soonawala D, Kroon FP, Adegnika AA, Deelder AM, Hokke CH, Yazdanbakhsh M, Wuhrer M. 2012. Changes in antigen-specific IgG1 Fc N-glycosylation upon influenza and tetanus vaccination. Mol Cell Proteomics 11:M111. doi:10.1074/mcp.M111.014563 PMC3322571 22184099

[28] Lofano G, Gorman MJ, Yousif AS, Yu W-H, Fox JM, Dugast A-S, Ackerman ME, Suscovich TJ, Weiner J, Barouch D, Streeck H, Little S, Smith D, Richman D, Lauffenburger D, Walker BD, Diamond MS, Alter G. 2018. Antigen-specific antibody Fc glycosylation enhances humoral immunity via the recruitment of complement. Sci Immunol 3:eaat7796. doi:10.1126/sciimmunol.aat7796 30120121 PMC6298214

[29] Francica JR, Zak DE, Linde C, Siena E, Johnson C, Juraska M, Yates NL, Gunn B, De Gregorio E, Flynn BJ, Valiante NM, Malyala P, Barnett SW, Sarkar P, Singh M, Jain S, Ackerman M, Alam M, Ferrari G, Salazar A, Tomaras GD, O’Hagan DT, Aderem A, Alter G, Seder RA. 2017. Innate transcriptional effects by adjuvants on the magnitude, quality, and durability of HIV envelope responses in NHPs. Blood Adv 1:2329–2342. doi:10.1182/bloodadvances.2017011411 29296883 PMC5729628

[30] Wang TT, Maamary J, Tan GS, Bournazos S, Davis CW, Krammer F, Schlesinger SJ, Palese P, Ahmed R, Ravetch JV. 2015. Anti-HA glycoforms drive B cell affinity selection and determine influenza vaccine efficacy. Cell 162:160–169. doi:10.1016/j.cell.2015.06.026 26140596 PMC4594835

[31] Mahan AE, Jennewein MF, Suscovich T, Dionne K, Tedesco J, Chung AW, Streeck H, Pau M, Schuitemaker H, Francis D, Fast P, Laufer D, Walker BD, Baden L, Barouch DH, Alter G, Trkola A. 2016. Antigen-specific antibody glycosylation is regulated via vaccination. PLoS Pathog 12:e1005456. doi:10.1371/journal.ppat.1005456 26982805 PMC4794126

[32] Maul RW, Gearhart PJ. 2010. AID and somatic hypermutation. Adv Immunol 105:159–191. doi:10.1016/S0065-2776(10)05006-6 20510733 PMC2954419

[33] Prakash S, Johnson RE, Prakash L. 2005. Eukaryotic translesion synthesis DNA polymerases: specificity of structure and function. Annu Rev Biochem 74:317–353. doi:10.1146/annurev.biochem.74.082803.133250 15952890

[34] Matsuda T, Bebenek K, Masutani C, Rogozin IB, Hanaoka F, Kunkel TA. 2001. Error rate and specificity of human and murine DNA polymerase eta. J Mol Biol 312:335–346. doi:10.1006/jmbi.2001.4937 11554790

[35] Pavlov YI, Rogozin IB, Galkin AP, Aksenova AY, Hanaoka F, Rada C, Kunkel TA. 2002. Correlation of somatic hypermutation specificity and A-T base pair substitution errors by DNA polymerase eta during copying of a mouse immunoglobulin kappa light chain transgene. Proc Natl Acad Sci U S A 99:9954–9959. doi:10.1073/pnas.152126799 12119399 PMC126606

[36] Di Noia JM, Neuberger MS. 2007. Molecular mechanisms of antibody somatic hypermutation. Annu Rev Biochem 76:1–22. doi:10.1146/annurev.biochem.76.061705.090740 17328676

[37] Lieber MR. 2010. The mechanism of double-strand DNA break repair by the nonhomologous DNA end-joining pathway. Annu Rev Biochem 79:181–211. doi:10.1146/annurev.biochem.052308.093131 20192759 PMC3079308

[38] Zhang Y, Zhang X, Dai HQ, Hu H, Alt FW. 2022. The role of chromatin loop extrusion in antibody diversification. Nat Rev Immunol 22:550–566. doi:10.1038/s41577-022-00679-3 35169260 PMC9376198

[39] Sela-Culang I, Kunik V, Ofran Y. 2013. The structural basis of antibody-antigen recognition. Front Immunol 4:302. doi:10.3389/fimmu.2013.00302 24115948 PMC3792396

[40] Chothia C, Lesk AM, Tramontano A, Levitt M, Smith-Gill SJ, Air G, Sheriff S, Padlan EA, Davies D, Tulip WR, Colman PM, Spinelli S, Alzari PM, Poljak RJ. 1989. Conformations of immunoglobulin hypervariable regions. Nature 342:877–883. doi:10.1038/342877a0 2687698

[41] Collis AVJ, Brouwer AP, Martin ACR. 2003. Analysis of the antigen combining site: correlations between length and sequence composition of the hypervariable loops and the nature of the antigen. J Mol Biol 325:337–354. doi:10.1016/s0022-2836(02)01222-6 12488099

[42] Feng Y, Seija N, Di Noia JM, Martin A. 2020. AID in antibody diversification: there and back again. Trends Immunol 41:586–600. doi:10.1016/j.it.2020.04.009 32434680 PMC7183997

[43] Wibmer CK, Moore PL, Morris L. 2015. HIV broadly neutralizing antibody targets. Curr Opin HIV AIDS 10:135–143. doi:10.1097/COH.0000000000000153 25760932 PMC4437463

[44] Andrade DV, Warnes C, Young E, Katzelnick LC, Balmaseda A, de Silva AM, Baric RS, Harris E. 2019. Tracking the polyclonal neutralizing antibody response to a dengue virus serotype 1 type-specific epitope across two populations in Asia and the Americas. Sci Rep 9:16258. doi:10.1038/s41598-019-52511-z 31700029 PMC6838341

[45] Bournazos S, Chow S-K, Abboud N, Casadevall A, Ravetch JV. 2014. Human IgG Fc domain engineering enhances antitoxin neutralizing antibody activity. J Clin Invest 124:725–729. doi:10.1172/JCI72676 24401277 PMC3904629

[46] Wenzel EV, Bosnak M, Tierney R, Schubert M, Brown J, Dübel S, Efstratiou A, Sesardic D, Stickings P, Hust M. 2020. Human antibodies neutralizing diphtheria toxin in vitro and in vivo. Sci Rep 10:571. doi:10.1038/s41598-019-57103-5 31953428 PMC6969050

[47] Bournazos S, Klein F, Pietzsch J, Seaman MS, Nussenzweig MC, Ravetch JV. 2014. Broadly neutralizing anti-HIV-1 antibodies require Fc effector functions for in vivo activity. Cell 158:1243–1253. doi:10.1016/j.cell.2014.08.023 25215485 PMC4167398

[48] Fox A, Mai LQ, Thanh LT, Wolbers M, Le Khanh Hang N, Thai PQ, Thi Thu Yen N, Minh Hoa LN, Bryant JE, Duong TN, Thoang DD, Barr IG, Wertheim H, Farrar J, Hien NT, Horby P. 2015. Hemagglutination inhibiting antibodies and protection against seasonal and pandemic influenza infection. J Infect 70:187–196. doi:10.1016/j.jinf.2014.09.003 25224643 PMC4309889

[49] Tumpey TM, García-Sastre A, Taubenberger JK, Palese P, Swayne DE, Pantin-Jackwood MJ, Schultz-Cherry S, Solórzano A, Van Rooijen N, Katz JM, Basler CF. 2005. Pathogenicity of influenza viruses with genes from the 1918 pandemic virus: functional roles of alveolar macrophages and neutrophils in limiting virus replication and mortality in mice. J Virol 79:14933–14944. doi:10.1128/JVI.79.23.14933-14944.2005 16282492 PMC1287592

[50] Brandenburg B, Koudstaal W, Goudsmit J, Klaren V, Tang C, Bujny MV, Korse H, Kwaks T, Otterstrom JJ, Juraszek J, van Oijen AM, Vogels R, Friesen RHE. 2013. Mechanisms of hemagglutinin targeted influenza virus neutralization. PLoS One 8:e80034. doi:10.1371/journal.pone.0080034 24348996 PMC3862845

[51] Ekiert DC, Friesen RHE, Bhabha G, Kwaks T, Jongeneelen M, Yu W, Ophorst C, Cox F, Korse H, Brandenburg B, Vogels R, Brakenhoff JPJ, Kompier R, Koldijk MH, Cornelissen L, Poon LLM, Peiris M, Koudstaal W, Wilson IA, Goudsmit J. 2011. A highly conserved neutralizing epitope on group 2 influenza A viruses. Science 333:843–850. doi:10.1126/science.1204839 21737702 PMC3210727

[52] He W, Tan GS, Mullarkey CE, Lee AJ, Lam MMW, Krammer F, Henry C, Wilson PC, Ashkar AA, Palese P, Miller MS. 2016. Epitope specificity plays a critical role in regulating antibody-dependent cell-mediated cytotoxicity against influenza A virus. Proc Natl Acad Sci U S A 113:11931–11936. doi:10.1073/pnas.1609316113 27698132 PMC5081650

[53] Rattan A, Pawar SD, Nawadkar R, Kulkarni N, Lal G, Mullick J, Sahu A, Thomas PG. 2017. Synergy between the classical and alternative pathways of complement is essential for conferring effective protection against the pandemic influenza A(H1N1) 2009 virus infection. PLoS Pathog 13:e1006248. doi:10.1371/journal.ppat.1006248 28301559 PMC5354441

[54] Getahun A, Cambier JC. 2015. Of ITIMs, ITAMs, and ITAMis: revisiting immunoglobulin Fc receptor signaling. Immunol Rev 268:66–73. doi:10.1111/imr.12336 26497513 PMC4621791

[55] Mayadas TN, Cullere X, Lowell CA. 2014. The multifaceted functions of neutrophils. Annu Rev Pathol 9:181–218. doi:10.1146/annurev-pathol-020712-164023 24050624 PMC4277181

[56] Aleyd E, van Hout MWM, Ganzevles SH, Hoeben KA, Everts V, Bakema JE, van Egmond M. 2014. IgA enhances NETosis and release of neutrophil extracellular traps by polymorphonuclear cells via Fcα receptor I. J Immunol 192:2374–2383. doi:10.4049/jimmunol.1300261 24493821

[57] Savina A, Amigorena S. 2007. Phagocytosis and antigen presentation in dendritic cells. Immunol Rev 219:143–156. doi:10.1111/j.1600-065X.2007.00552.x 17850487

[58] Nepomuceno RR, Tenner AJ. 1998. C1qR_p_, the C1q receptor that enhances phagocytosis, is detected specifically in human cells of myeloid lineage, endothelial cells, and platelets. J Immunol 160:1929–1935. 9469455

[59] Ross GD, Newman SL, Lambris JD, Devery-Pocius JE, Cain JA, Lachmann PJ. 1983. Generation of three different fragments of bound C3 with purified factor I or serum. II. Location of binding sites in the C3 fragments for factors B and H, complement receptors, and bovine conglutinin. J Exp Med 158:334–352. doi:10.1084/jem.158.2.334 6224880 PMC2187331

[60] Jennewein MF, Kosikova M, Noelette FJ, Radvak P, Boudreau CM, Campbell JD, Chen WH, Xie H, Alter G, Pasetti MF. 2022. Functional and structural modifications of influenza antibodies during pregnancy. iScience 25:104088. doi:10.1016/j.isci.2022.104088 35402869 PMC8991102

[61] Leitner WW, Haraway M, Pierson T, Bergmann-Leitner ES. 2020. Role of opsonophagocytosis in immune protection against malaria. Vaccines (Basel) 8:264. doi:10.3390/vaccines8020264 32486320 PMC7350021

[62] Kwon Y-C, Kim H, Meyer K, Di Bisceglie AM, Ray R. 2016. Distinct CD55 isoform synthesis and inhibition of complement-dependent cytolysis by hepatitis C virus. J Immunol 197:1127–1136. doi:10.4049/jimmunol.1600631 27357152 PMC4976035

[63] Wang G, de Jong RN, van den Bremer ETJ, Beurskens FJ, Labrijn AF, Ugurlar D, Gros P, Schuurman J, Parren PWHI, Heck AJR. 2016. Molecular basis of assembly and activation of complement component C1 in complex with immunoglobulin G1 and antigen. Mol Cell 63:135–145. doi:10.1016/j.molcel.2016.05.016 27320199

[64] Huber G, Bánki Z, Lengauer S, Stoiber H. 2011. Emerging role for complement in HIV infection. Curr Opin HIV AIDS 6:419–426. doi:10.1097/COH.0b013e3283495a26 21825871

[65] Dunkelberger JR, Song W-C. 2010. Complement and its role in innate and adaptive immune responses. Cell Res 20:34–50. doi:10.1038/cr.2009.139 20010915

[66] Fernández FJ, Santos-López J, Martínez-Barricarte R, Querol-García J, Martín-Merinero H, Navas-Yuste S, Savko M, Shepard WE, Rodríguez de Córdoba S, Vega MC. 2022. The crystal structure of iC3b-CR3 αI reveals a modular recognition of the main opsonin iC3b by the CR3 integrin receptor. Nat Commun 13:1955. doi:10.1038/s41467-022-29580-2 35413960 PMC9005620

[67] van Lookeren Campagne M, Wiesmann C, Brown EJ. 2007. Macrophage complement receptors and pathogen clearance. Cell Microbiol 9:2095–2102. doi:10.1111/j.1462-5822.2007.00981.x 17590164

[68] van Lookeren Campagne M, Verschoor A. 2018. Pathogen clearance and immune adherence "revisited": immuno-regulatory roles for CRIg. Semin Immunol 37:4–11. doi:10.1016/j.smim.2018.02.007 29573978

[69] Wang Y, Zhang H, He YW. 2019. The complement receptors C3aR and C5aR are a new class of immune checkpoint receptor in cancer immunotherapy. Front Immunol 10:1574. doi:10.3389/fimmu.2019.01574 31379815 PMC6658873

[70] Feng Y, Zhao C, Deng Y, Wang H, Ma L, Liu S, Tian X, Wang B, Bin Y, Chen P, Yan W, Fu P, Shao Z. 2023. Mechanism of activation and biased signaling in complement receptor C5aR1. Cell Res 33:312–324. doi:10.1038/s41422-023-00779-2 36806352 PMC9937529

[71] Skokowa J, Ali SR, Felda O, Kumar V, Konrad S, Shushakova N, Schmidt RE, Piekorz RP, Nürnberg B, Spicher K, Birnbaumer L, Zwirner J, Claassens JWC, Verbeek JS, van Rooijen N, Köhl J, Gessner JE. 2005. Macrophages induce the inflammatory response in the pulmonary Arthus reaction through G alpha i2 activation that controls C5aR and Fc receptor cooperation. J Immunol 174:3041–3050. doi:10.4049/jimmunol.174.5.3041 15728518

[72] Li XX, Lee JD, Kemper C, Woodruff TM. 2019. The complement receptor C5aR2: a powerful modulator of innate and adaptive immunity. J Immunol 202:3339–3348. doi:10.4049/jimmunol.1900371 31160390

[73] Platonov AE, Vershinina IV, Käyhty H, Fijen CAP, Würzner R, Kuijper EJ. 2003. Antibody-dependent killing of meningococci by human neutrophils in serum of late complement component-deficient patients. Int Arch Allergy Immunol 130:314–321. doi:10.1159/000070219 12740533

[74] Singh S, Uppuluri P, Mamouei Z, Alqarihi A, Elhassan H, French S, Lockhart SR, Chiller T, Edwards JE, Ibrahim AS, Gaffen SL. 2019. The NDV-3A vaccine protects mice from multidrug resistant Candida auris infection. PLoS Pathog 15:e1007460. doi:10.1371/journal.ppat.1007460 31381597 PMC6695204

[75] Mullarkey CE, Bailey MJ, Golubeva DA, Tan GS, Nachbagauer R, He W, Novakowski KE, Bowdish DM, Miller MS, Palese P. 2016. Broadly neutralizing hemagglutinin stalk-specific antibodies induce potent phagocytosis of immune complexes by neutrophils in an Fc-dependent manner. mBio 7:e01624-16. doi:10.1128/mBio.01624-16 27703076 PMC5050345

[76] van den Broek B, van Els C, Kuipers B, van Aerde K, Henriet SS, de Groot R, de Jonge MI, Langereis JD, van der Flier M. 2019. Multi-component meningococcal serogroup B (MenB)-4C vaccine induces effective opsonophagocytic killing in children with a complement deficiency. Clin Exp Immunol 198:381–389. doi:10.1111/cei.13368 31487400 PMC6857189

[77] Kennedy AD, Willment JA, Dorward DW, Williams DL, Brown GD, DeLeo FR. 2007. Dectin-1 promotes fungicidal activity of human neutrophils. Eur J Immunol 37:467–478. doi:10.1002/eji.200636653 17230442

[78] Barouch DH, Alter G, Broge T, Linde C, Ackerman ME, Brown EP, Borducchi EN, Smith KM, Nkolola JP, Liu J, et al.. 2015. Protective efficacy of adenovirus/protein vaccines against SIV challenges in rhesus monkeys. Science 349:320–324. doi:10.1126/science.aab3886 26138104 PMC4653134

[79] Ackerman ME, Mikhailova A, Brown EP, Dowell KG, Walker BD, Bailey-Kellogg C, Suscovich TJ, Alter G. 2016. Polyfunctional HIV-specific antibody responses are associated with spontaneous HIV control. PLoS Pathog 12:e1005315. doi:10.1371/journal.ppat.1005315 26745376 PMC4706315

[80] Forthal DN, Landucci G, Bream J, Jacobson LP, Phan TB, Montoya B. 2007. FcγRIIa genotype predicts progression of HIV infection. J Immunol 179:7916–7923. doi:10.4049/jimmunol.179.11.7916 18025239

[81] Gunn BM, Lu R, Slein MD, Ilinykh PA, Huang K, Atyeo C, Schendel SL, Kim J, Cain C, Roy V, Suscovich TJ, Takada A, Halfmann PJ, Kawaoka Y, Pauthner MG, Momoh M, Goba A, Kanneh L, Andersen KG, Schieffelin JS, Grant D, Garry RF, Saphire EO, Bukreyev A, Alter G. 2021. A Fc engineering approach to define functional humoral correlates of immunity against Ebola virus. Immunity 54:815–828. doi:10.1016/j.immuni.2021.03.009 33852832 PMC8111768

[82] Saphire EO, Schendel SL, Gunn BM, Milligan JC, Alter G. 2018. Antibody-mediated protection against Ebola virus. Nat Immunol 19:1169–1178. doi:10.1038/s41590-018-0233-9 30333617 PMC6814399

[83] Pallesen J, Murin CD, de Val N, Cottrell CA, Hastie KM, Turner HL, Fusco ML, Flyak AI, Zeitlin L, Crowe JE, Andersen KG, Saphire EO, Ward AB. 2016. Structures of Ebola virus GP and sGP in complex with therapeutic antibodies. Nat Microbiol 1:16128. doi:10.1038/nmicrobiol.2016.128 27562261 PMC5003320

[84] Henao-Restrepo AM, Camacho A, Longini IM, Watson CH, Edmunds WJ, Egger M, Carroll MW, Dean NE, Diatta I, Doumbia M, et al.. 2017. Efficacy and effectiveness of an rVSV-vectored vaccine in preventing Ebola virus disease: final results from the Guinea ring vaccination, open-label, cluster-randomised trial (Ebola Ça Suffit!). Lancet 389:505–518. doi:10.1016/S0140-6736(16)32621-6 28017403 PMC5364328

[85] Mackin SR, Desai P, Whitener BM, Karl CE, Liu M, Baric RS, Edwards DK, Chicz TM, McNamara RP, Alter G, Diamond MS. 2023. Fc-γR-dependent antibody effector functions are required for vaccine-mediated protection against antigen-shifted variants of SARS-CoV-2. Nat Microbiol 8:569–580. doi:10.1038/s41564-023-01359-1 37012355 PMC10797606

[86] Adams LE, Leist SR, Dinnon KH, West A, Gully KL, Anderson EJ, Loome JF, Madden EA, Powers JM, Schäfer A, Sarkar S, Castillo IN, Maron JS, McNamara RP, Bertera HL, Zweigert MR, Higgins JS, Hampton BK, Premkumar L, Alter G, Montgomery SA, Baxter VK, Heise MT, Baric RS. 2023. Fc-mediated pan-sarbecovirus protection after alphavirus vector vaccination. Cell Rep 42:112326. doi:10.1016/j.celrep.2023.112326 37000623 PMC10063157

[87] Kaplonek P, Cizmeci D, Fischinger S, Collier A-R, Suscovich T, Linde C, Broge T, Mann C, Amanat F, Dayal D, Rhee J, de St Aubin M, Nilles EJ, Musk ER, Menon AS, Saphire EO, Krammer F, Lauffenburger DA, Barouch DH, Alter G. 2022. mRNA-1273 and BNT162b2 COVID-19 vaccines elicit antibodies with differences in Fc-mediated effector functions. Sci Transl Med 14:eabm2311. doi:10.1126/scitranslmed.abm2311 35348368 PMC8995030

[88] Beaudoin-Bussières G, Chen Y, Ullah I, Prévost J, Tolbert WD, Symmes K, Ding S, Benlarbi M, Gong SY, Tauzin A, et al.. 2022. A Fc-enhanced NTD-binding non-neutralizing antibody delays virus spread and synergizes with a nAb to protect mice from lethal SARS-CoV-2 infection. Cell Rep 38:110368. doi:10.1016/j.celrep.2022.110368 35123652 PMC8786652

[89] Bowman KA, Stein D, Shin S, Ferbas KG, Tobin NH, Mann C, Fischinger S, Ollmann Saphire E, Lauffenburger D, Rimoin AW, Aldrovandi G, Alter G. 2022. Hybrid immunity shifts the Fc-effector quality of SARS-CoV-2 mRNA vaccine-induced immunity. mBio 13:e0164722. doi:10.1128/mbio.01647-22 36000735 PMC9600672

[90] Garcia-Beltran WF, Lam EC, Astudillo MG, Yang D, Miller TE, Feldman J, Hauser BM, Caradonna TM, Clayton KL, Nitido AD, Murali MR, Alter G, Charles RC, Dighe A, Branda JA, Lennerz JK, Lingwood D, Schmidt AG, Iafrate AJ, Balazs AB. 2021. COVID-19-neutralizing antibodies predict disease severity and survival. Cell 184:476–488. doi:10.1016/j.cell.2020.12.015 33412089 PMC7837114

[91] Zohar T, Loos C, Fischinger S, Atyeo C, Wang C, Slein MD, Burke J, Yu J, Feldman J, Hauser BM, Caradonna T, Schmidt AG, Cai Y, Streeck H, Ryan ET, Barouch DH, Charles RC, Lauffenburger DA, Alter G. 2020. Compromised humoral functional evolution tracks with SARS-CoV-2 mortality. Cell 183:1508–1519. doi:10.1016/j.cell.2020.10.052 33207184 PMC7608014

[92] Suscovich TJ, Fallon JK, Das J, Demas AR, Crain J, Linde CH, Michell A, Natarajan H, Arevalo C, Broge T, et al.. 2020. Mapping functional humoral correlates of protection against malaria challenge following RTS,S/AS01 vaccination. Sci Transl Med 12:eabb4757. doi:10.1126/scitranslmed.abb4757 32718991

[93] Regules JA, Cicatelli SB, Bennett JW, Paolino KM, Twomey PS, Moon JE, Kathcart AK, Hauns KD, Komisar JL, Qabar AN, et al.. 2016. Fractional third and fourth dose of RTS,S/AS01 malaria candidate vaccine: a phase 2a controlled human malaria parasite infection and immunogenicity study. J Infect Dis 214:762–771. doi:10.1093/infdis/jiw237 27296848

[94] RTS,S Clinical Trials Partnership. 2015. Efficacy and safety of RTS,S/AS01 malaria vaccine with or without a booster dose in infants and children in Africa: final results of a phase 3, individually randomised, controlled trial. Lancet 386:31–45. doi:10.1016/S0140-6736(15)60721-8 25913272 PMC5626001

[95] Lohse S, Loew S, Kretschmer A, Jansen JHM, Meyer S, Ten Broeke T, Rösner T, Dechant M, Derer S, Klausz K, Kellner C, Schwanbeck R, French RR, Tipton TRW, Cragg MS, Schewe DM, Peipp M, Leusen JHW, Valerius T. 2018. Effector mechanisms of IgA antibodies against CD20 include recruitment of myeloid cells for antibody-dependent cell-mediated cytotoxicity and complement-dependent cytotoxicity. Br J Haematol 181:413–417. doi:10.1111/bjh.14624 28449349

[96] Karagiannis SN, Josephs DH, Karagiannis P, Gilbert AE, Saul L, Rudman SM, Dodev T, Koers A, Blower PJ, Corrigan C, Beavil AJ, Spicer JF, Nestle FO, Gould HJ. 2012. Recombinant IgE antibodies for passive immunotherapy of solid tumours: from concept towards clinical application. Cancer Immunol Immunother 61:1547–1564. doi:10.1007/s00262-011-1162-8 22139135 PMC11028906

[97] Jin BR, Kim SJ, Lee JM, Kang SH, Han HJ, Jang YS, Seo GY, Kim PH. 2013. Alum directly modulates murine B lymphocytes to produce IgG1 Isotype. Immune Netw 13:10–15. doi:10.4110/in.2013.13.1.10 23559895 PMC3607705

[98] He X, Liang H, Hong K, Li H, Peng H, Zhao Y, Jia M, Ruan Y, Shao Y. 2013. The potential role of CD16+ Vγ2Vδ2 T cell-mediated antibody-dependent cell-mediated cytotoxicity in control of HIV type 1 disease. AIDS Res Hum Retroviruses 29:1562–1570. doi:10.1089/AID.2013.0111 23957587 PMC3848486

[99] Vanderven HA, Liu L, Ana-Sosa-Batiz F, Nguyen TH, Wan Y, Wines B, Hogarth PM, Tilmanis D, Reynaldi A, Parsons MS, Hurt AC, Davenport MP, Kotsimbos T, Cheng AC, Kedzierska K, Zhang X, Xu J, Kent SJ. 2017. Fc functional antibodies in humans with severe H7N9 and seasonal influenza. JCI Insight 2:e92750. doi:10.1172/jci.insight.92750 28679958 PMC5499372

[100] Larrañaga CL, Ampuero SL, Luchsinger VF, Carrión FA, Aguilar NV, Morales PR, Palomino MAM, Tapia LF, Avendaño LF. 2009. Impaired immune response in severe human lower tract respiratory infection by respiratory syncytial virus. Pediatr Infect Dis J 28:867–873. doi:10.1097/INF.0b013e3181a3ea71 19738511

[101] Saphire EO, Schendel SL, Fusco ML, Gangavarapu K, Gunn BM, Wec AZ, Halfmann PJ, Brannan JM, Herbert AS, Qiu X, et al.. 2018. Systematic analysis of monoclonal antibodies against Ebola virus GP defines features that contribute to protection. Cell 174:938–952. doi:10.1016/j.cell.2018.07.033 30096313 PMC6102396

[102] DiLillo DJ, Tan GS, Palese P, Ravetch JV. 2014. Broadly neutralizing hemagglutinin stalk-specific antibodies require FcγR interactions for protection against influenza virus in vivo. Nat Med 20:143–151. doi:10.1038/nm.3443 24412922 PMC3966466

[103] Garand M, Goodier M, Owolabi O, Donkor S, Kampmann B, Sutherland JS. 2018. Functional and phenotypic changes of natural killer cells in whole blood during Mycobacterium tuberculosis infection and disease. Front Immunol 9:257. doi:10.3389/fimmu.2018.00257 29520269 PMC5827559

[104] Lu LL, Chung AW, Rosebrock TR, Ghebremichael M, Yu WH, Grace PS, Schoen MK, Tafesse F, Martin C, Leung V, Mahan AE, Sips M, Kumar MP, Tedesco J, Robinson H, Tkachenko E, Draghi M, Freedberg KJ, Streeck H, Suscovich TJ, Lauffenburger DA, Restrepo BI, Day C, Fortune SM, Alter G. 2016. A functional role for antibodies in tuberculosis. Cell 167:433–443. doi:10.1016/j.cell.2016.08.072 27667685 PMC5526202

[105] Chen Q, Amaladoss A, Ye W, Liu M, Dummler S, Kong F, Wong LH, Loo HL, Loh E, Tan SQ, Tan TC, Chang KTE, Dao M, Suresh S, Preiser PR, Chen J. 2014. Human natural killer cells control Plasmodium falciparum infection by eliminating infected red blood cells. Proc Natl Acad Sci U S A 111:1479–1484. doi:10.1073/pnas.1323318111 24474774 PMC3910619

[106] White MT, Bejon P, Olotu A, Griffin JT, Riley EM, Kester KE, Ockenhouse CF, Ghani AC. 2013. The relationship between RTS,S vaccine-induced antibodies, CD4^+^ T cell responses and protection against Plasmodium falciparum infection. PLoS One 8:e61395. doi:10.1371/journal.pone.0061395 23613845 PMC3628884

[107] Lieke T, Steeg C, Graefe SEB, Fleischer B, Jacobs T. 2006. Interaction of natural killer cells with Trypanosoma cruzi-infected fibroblasts. Clin Exp Immunol 145:357–364. doi:10.1111/j.1365-2249.2006.03118.x 16879257 PMC1809687

[108] Ricklin D, Hajishengallis G, Yang K, Lambris JD. 2010. Complement: a key system for immune surveillance and homeostasis. Nat Immunol 11:785–797. doi:10.1038/ni.1923 20720586 PMC2924908

[109] Saifuddin M, Hedayati T, Atkinson JP, Holguin MH, Parker CJ, Spear GT. 1997. Human immunodeficiency virus type 1 incorporates both glycosyl phosphatidylinositol-anchored CD55 and CD59 and integral membrane CD46 at levels that protect from complement-mediated destruction. J Gen Virol 78 ( Pt 8):1907–1911. doi:10.1099/0022-1317-78-8-1907 9266986

[110] Vanderplasschen A, Mathew E, Hollinshead M, Sim RB, Smith GL. 1998. Extracellular enveloped vaccinia virus is resistant to complement because of incorporation of host complement control proteins into its envelope. Proc Natl Acad Sci U S A 95:7544–7549. doi:10.1073/pnas.95.13.7544 9636186 PMC22678

[111] Benhnia M-E-I, McCausland MM, Laudenslager J, Granger SW, Rickert S, Koriazova L, Tahara T, Kubo RT, Kato S, Crotty S. 2009. Heavily isotype-dependent protective activities of human antibodies against vaccinia virus extracellular virion antigen B5. J Virol 83:12355–12367. doi:10.1128/JVI.01593-09 19793826 PMC2786738

[112] Jang MS, Sahastrabuddhe S, Yun C-H, Han SH, Yang JS. 2016. Serum bactericidal assay for the evaluation of typhoid vaccine using a semi-automated colony-counting method. Microb Pathog 97:19–26. doi:10.1016/j.micpath.2016.05.013 27216239 PMC4944902

[113] Nahm MH, Yu J, Weerts HP, Wenzel H, Tamilselvi CS, Chandrasekaran L, Pasetti MF, Mani S, Kaminski RW. 2018. Development, interlaboratory evaluations, and application of a simple, high-throughput Shigella serum bactericidal assay. mSphere 3:e00146-18. doi:10.1128/mSphere.00146-18 29898979 PMC6001606

[114] Mehlhop E, Diamond MS. 2006. Protective immune responses against West Nile virus are primed by distinct complement activation pathways. J Exp Med 203:1371–1381. doi:10.1084/jem.20052388 16651386 PMC2121216

[115] Huber M, Fischer M, Misselwitz B, Manrique A, Kuster H, Niederöst B, Weber R, von Wyl V, Günthard HF, Trkola A. 2006. Complement lysis activity in autologous plasma is associated with lower viral loads during the acute phase of HIV-1 infection. PLoS Med 3:e441. doi:10.1371/journal.pmed.0030441 17121450 PMC1637124

[116] Benhnia M-E-I, McCausland MM, Moyron J, Laudenslager J, Granger S, Rickert S, Koriazova L, Kubo R, Kato S, Crotty S. 2009. Vaccinia virus extracellular enveloped virion neutralization in vitro and protection in vivo depend on complement. J Virol 83:1201–1215. doi:10.1128/JVI.01797-08 19019965 PMC2620895

[117] Blander JM, Barbet G. 2018. Exploiting vita-PAMPs in vaccines. Curr Opin Pharmacol 41:128–136. doi:10.1016/j.coph.2018.05.012 29890457 PMC6110613

[118] Mosca F, Tritto E, Muzzi A, Monaci E, Bagnoli F, Iavarone C, O’Hagan D, Rappuoli R, De Gregorio E. 2008. Molecular and cellular signatures of human vaccine adjuvants. Proc Natl Acad Sci U S A 105:10501–10506. doi:10.1073/pnas.0804699105 18650390 PMC2483233

[119] Morel S, Didierlaurent A, Bourguignon P, Delhaye S, Baras B, Jacob V, Planty C, Elouahabi A, Harvengt P, Carlsen H, Kielland A, Chomez P, Garçon N, Van Mechelen M. 2011. Adjuvant system AS03 containing α-tocopherol modulates innate immune response and leads to improved adaptive immunity. Vaccine 29:2461–2473. doi:10.1016/j.vaccine.2011.01.011 21256188

[120] Sato R, Reuter T, Hiranuma R, Shibata T, Fukui R, Motoi Y, Murakami Y, Tsukamoto H, Yamazaki S, Liu K, Saitoh S-I, Latz E, Miyake K. 2020. The impact of cell maturation and tissue microenvironments on the expression of endosomal toll-like receptors in monocytes and macrophages. Int Immunol 32:785–798. doi:10.1093/intimm/dxaa055 32840578

[121] Hu W, Pasare C. 2013. Location, location, location: tissue-specific regulation of immune responses. J Leukoc Biol 94:409–421. doi:10.1189/jlb.0413207 23825388 PMC3747123

[122] Boyaka PN. 2017. Inducing mucosal IgA: a challenge for vaccine adjuvants and delivery systems. J Immunol 199:9–16. doi:10.4049/jimmunol.1601775 28630108 PMC5719502

[123] Kenney RT, Yu J, Guebre-Xabier M, Frech SA, Lambert A, Heller BA, Ellingsworth LR, Eyles JE, Williamson ED, Glenn GM. 2004. Induction of protective immunity against lethal anthrax challenge with a patch. J Infect Dis 190:774–782. doi:10.1086/422694 15272406

[124] Koutsonanos DG, del Pilar Martin M, Zarnitsyn VG, Jacob J, Prausnitz MR, Compans RW, Skountzou I. 2011. Serological memory and long-term protection to novel H1N1 influenza virus after skin vaccination. J Infect Dis 204:582–591. doi:10.1093/infdis/jir094 21685355 PMC3144165

[125] Sullivan SP, Koutsonanos DG, Del Pilar Martin M, Lee JW, Zarnitsyn V, Choi SO, Murthy N, Compans RW, Skountzou I, Prausnitz MR. 2010. Dissolving polymer microneedle patches for influenza vaccination. Nat Med 16:915–920. doi:10.1038/nm.2182 20639891 PMC2917494

[126] Koutsonanos DG, Esser ES, McMaster SR, Kalluri P, Lee J-W, Prausnitz MR, Skountzou I, Denning TL, Kohlmeier JE, Compans RW. 2015. Enhanced immune responses by skin vaccination with influenza subunit vaccine in young hosts. Vaccine 33:4675–4682. doi:10.1016/j.vaccine.2015.01.086 25744228 PMC5757502

[127] Boonnak K, Dhitavat J, Thantamnu N, Kosoltanapiwat N, Auayporn M, Jiang L, Puthavathana P, Pitisuttithum P. 2017. Immune responses to intradermal and intramuscular inactivated influenza vaccine among older age group. Vaccine 35:7339–7346. doi:10.1016/j.vaccine.2017.10.106 29157960

[128] Seder RA, Chang L-J, Enama ME, Zephir KL, Sarwar UN, Gordon IJ, Holman LA, James ER, Billingsley PF, Gunasekera A, et al.. 2013. Protection against malaria by intravenous immunization with a nonreplicating sporozoite vaccine. Science 341:1359–1365. doi:10.1126/science.1241800 23929949

[129] Darrah PA, Zeppa JJ, Maiello P, Hackney JA, Wadsworth MH II, Hughes TK, Pokkali S, Swanson PA II, Grant NL, Rodgers MA, Kamath M, Causgrove CM, Laddy DJ, Bonavia A, Casimiro D, Lin PL, Klein E, White AG, Scanga CA, Shalek AK, Roederer M, Flynn JL, Seder RA. 2020. Prevention of tuberculosis in macaques after intravenous BCG immunization. Nature 577:95–102. doi:10.1038/s41586-019-1817-8 31894150 PMC7015856

[130] Bartsch YC, St Denis KJ, Kaplonek P, Kang J, Lam EC, Burns MD, Farkas EJ, Davis JP, Boribong BP, Edlow AG, Fasano A, Shreffler WG, Zavadska D, Johnson M, Goldblatt D, Balazs AB, Yonker LM, Alter G. 2022. SARS-CoV-2 mRNA vaccination elicits robust antibody responses in children. Sci Transl Med 14:eabn9237. doi:10.1126/scitranslmed.abn9237 35881018 PMC9348753

[131] Atyeo C, DeRiso EA, Davis C, Bordt EA, De Guzman RM, Shook LL, Yonker LM, Fasano A, Akinwunmi B, Lauffenburger DA, Elovitz MA, Gray KJ, Edlow AG, Alter G. 2021. COVID-19 mRNA vaccines drive differential antibody Fc-functional profiles in pregnant, lactating, and nonpregnant women. Sci Transl Med 13:eabi8631. doi:10.1126/scitranslmed.abi8631 34664972 PMC9067624

[132] Gunn BM, McNamara RP, Wood L, Taylor S, Devadhasan A, Guo W, Das J, Nilsson A, Shurtleff A, Dubey S, Eichberg M, Suscovich TJ, Saphire EO, Lauffenburger D, Coller B-A, Simon JK, Alter G. 2023. Antibodies against the Ebola virus soluble glycoprotein are associated with long-term vaccine-mediated protection of non-human primates. Cell Rep 42:112402. doi:10.1016/j.celrep.2023.112402 37061918 PMC10576837

[133] Bartsch YC, Tong X, Kang J, Avendaño MJ, Serrano EF, García-Salum T, Pardo-Roa C, Riquelme A, Cai Y, Renzi I, Stewart-Jones G, Chen B, Medina RA, Alter G. 2022. Omicron variant spike-specific antibody binding and Fc activity are preserved in recipients of mRNA or inactivated COVID-19 vaccines. Sci Transl Med 14:eabn9243. doi:10.1126/scitranslmed.abn9243 35289637 PMC8995028

[134] Gorman MJ, Patel N, Guebre-Xabier M, Zhu AL, Atyeo C, Pullen KM, Loos C, Goez-Gazi Y, Carrion R, Tian JH, et al.. 2021. Fab and Fc contribute to maximal protection against SARS-CoV-2 following NVX-CoV2373 subunit vaccine with matrix-M vaccination. Cell Rep Med 2:100405. doi:10.1016/j.xcrm.2021.100405 34485950 PMC8405506

[135] Francica JR, Flynn BJ, Foulds KE, Noe AT, Werner AP, Moore IN, Gagne M, Johnston TS, Tucker C, Davis RL, et al.. 2021. Protective antibodies elicited by SARS-CoV-2 spike protein vaccination are boosted in the lung after challenge in nonhuman primates. Sci Transl Med 13. doi:10.1126/scitranslmed.abi4547 PMC9266840 34315825

[136] Hassan AO, Shrihari S, Gorman MJ, Ying B, Yuan D, Raju S, Chen RE, Dmitriev IP, Kashentseva E, Adams LJ, Mann C, Davis-Gardner ME, Suthar MS, Shi P-Y, Saphire EO, Fremont DH, Curiel DT, Alter G, Diamond MS. 2021. An intranasal vaccine durably protects against SARS-CoV-2 variants in mice. Cell Rep 36:109452. doi:10.1016/j.celrep.2021.109452 34289385 PMC8270739

[137] Kasturi SP, Skountzou I, Albrecht RA, Koutsonanos D, Hua T, Nakaya HI, Ravindran R, Stewart S, Alam M, Kwissa M, Villinger F, Murthy N, Steel J, Jacob J, Hogan RJ, García-Sastre A, Compans R, Pulendran B. 2011. Programming the magnitude and persistence of antibody responses with innate immunity. Nature 470:543–547. doi:10.1038/nature09737 21350488 PMC3057367

[138] Ko E-J, Lee Y-T, Kim K-H, Jung Y-J, Lee Y, Denning TL, Kang S-M. 2016. Effects of MF59 adjuvant on induction of isotype-switched IgG antibodies and protection after immunization with T-dependent influenza virus vaccine in the absence of CD4^+^ T cells. J Virol 90:6976–6988. doi:10.1128/JVI.00339-16 27226368 PMC4944285

[139] Luo K, Gordy JT, Zavala F, Markham RB. 2020. A vaccine composed of antigen fused to a ligand for a receptor on immature dendritic cells elicits elevated antibody responses to malaria sporozoites in infant macaques. bioRxiv. doi:10.1101/530840 PMC7807052 33441615

[140] Boudreau CM, Yu WH, Suscovich TJ, Talbot HK, Edwards KM, Alter G. 2020. Selective induction of antibody effector functional responses using MF59-adjuvanted vaccination. J Clin Invest 130:662–672. doi:10.1172/JCI129520 31845904 PMC6994146

[141] Corbett KS, Nason MC, Flach B, Gagne M, O’Connell S, Johnston TS, Shah SN, Edara VV, Floyd K, Lai L, et al.. 2021. Immune correlates of protection by mRNA-1273 vaccine against SARS-CoV-2 in nonhuman primates. Science 373:eabj0299. doi:10.1126/science.abj0299 34529476 PMC8449013

[142] de Bruyn G, Wang J, Purvis A, Ruiz MS, Adhikarla H, Alvi S, Bonaparte MI, Brune D, Bueso A, Canter RM, et al.. 2022. Safety and immunogenicity of a variant-adapted SARS-CoV-2 recombinant protein vaccine with AS03 adjuvant as a booster in adults primed with authorized vaccines. Cold Spring Harbor Laboratory, MedRXiv. doi:10.1101/2022.12.02.22282931 PMC10391925 37533419

[143] Pavot V, Berry C, Kishko M, Anosova NG, Huang D, Tibbitts T, Raillard A, Gautheron S, Gutzeit C, Koutsoukos M, Chicz RM, Lecouturier V. 2022. Protein-based SARS-CoV-2 spike vaccine booster increases cross-neutralization against SARS-CoV-2 variants of concern in non-human primates. Nat Commun 13:1699. doi:10.1038/s41467-022-29219-2 35361754 PMC8971430

[144] Kumar S, Anselmo AC, Banerjee A, Zakrewsky M, Mitragotri S. 2015. Shape and size-dependent immune response to antigen-carrying nanoparticles. J Control Release 220:141–148. doi:10.1016/j.jconrel.2015.09.069 26437263

[145] Slifka MK, Amanna IJ. 2019. Role of multivalency and antigenic threshold in generating protective antibody responses. Front Immunol 10:956. doi:10.3389/fimmu.2019.00956 31118935 PMC6504826

[146] Dintzis HM, Dintzis RZ, Vogelstein B. 1976. Molecular determinants of immunogenicity: the immunon model of immune response. Proc Natl Acad Sci U S A 73:3671–3675. doi:10.1073/pnas.73.10.3671 62364 PMC431180

[147] Kanekiyo M, Joyce MG, Gillespie RA, Gallagher JR, Andrews SF, Yassine HM, Wheatley AK, Fisher BE, Ambrozak DR, Creanga A, et al.. 2019. Mosaic nanoparticle display of diverse influenza virus hemagglutinins elicits broad B cell responses. Nat Immunol 20:362–372. doi:10.1038/s41590-019-0395-0 30742080 PMC6380945

[148] Moon JJ, Suh H, Polhemus ME, Ockenhouse CF, Yadava A, Irvine DJ. 2012. Antigen-displaying lipid-enveloped PLGA nanoparticles as delivery agents for a Plasmodium vivax malaria vaccine. PLoS One 7:e31472. doi:10.1371/journal.pone.0031472 22328935 PMC3273465

[149] Mehrabi M, Montazeri H, Mohamadpour Dounighi N, Rashti A, Vakili-Ghartavol R. 2018. Chitosan-based nanoparticles in mucosal vaccine delivery. Arch Razi Inst 73:165–176. doi:10.22092/ari.2017.109235.1101 30280836

[150] Cohen AA, van Doremalen N, Greaney AJ, Andersen H, Sharma A, Starr TN, Keeffe JR, Fan C, Schulz JE, Gnanapragasam PNP, Kakutani LM, West AP, Saturday G, Lee YE, Gao H, Jette CA, Lewis MG, Tan TK, Townsend AR, Bloom JD, Munster VJ, Bjorkman PJ. 2022. Mosaic RBD nanoparticles protect against challenge by diverse sarbecoviruses in animal models. Science 377:eabq0839. doi:10.1126/science.abq0839 35857620 PMC9273039

[151] Cohen AA, Gnanapragasam PNP, Lee YE, Hoffman PR, Ou S, Kakutani LM, Keeffe JR, Wu H-J, Howarth M, West AP, Barnes CO, Nussenzweig MC, Bjorkman PJ. 2021. Mosaic nanoparticles elicit cross-reactive immune responses to zoonotic coronaviruses in mice. Science 371:735–741. doi:10.1126/science.abf6840 33436524 PMC7928838

[152] Varela-Aramburu S, Ghosh C, Goerdeler F, Priegue P, Moscovitz O, Seeberger PH. 2020. Targeting and inhibiting Plasmodium falciparum using ultra-small gold nanoparticles. ACS Appl Mater Interfaces 12:43380–43387. doi:10.1021/acsami.0c09075 32875786 PMC7586288

[153] Xu L, Liu Y, Chen Z, Li W, Liu Y, Wang L, Liu Y, Wu X, Ji Y, Zhao Y, Ma L, Shao Y, Chen C. 2012. Surface-engineered gold nanorods: promising DNA vaccine adjuvant for HIV-1 treatment. Nano Lett 12:2003–2012. doi:10.1021/nl300027p 22372996

[154] Carvalho GC, Sábio RM, de Cássia Ribeiro T, Monteiro AS, Pereira DV, Ribeiro SJL, Chorilli M. 2020. Highlights in mesoporous silica nanoparticles as a multifunctional controlled drug delivery nanoplatform for infectious diseases treatment. Pharm Res 37:191. doi:10.1007/s11095-020-02917-6 32895867 PMC7476752

[155] Gottardi R, Douradinha B. 2013. Carbon nanotubes as a novel tool for vaccination against infectious diseases and cancer. J Nanobiotechnology 11:30. doi:10.1186/1477-3155-11-30 24025216 PMC3846653

[156] Perrie Y, Crofts F, Devitt A, Griffiths HR, Kastner E, Nadella V. 2016. Designing liposomal adjuvants for the next generation of vaccines. Adv Drug Deliv Rev 99:85–96. doi:10.1016/j.addr.2015.11.005 26576719

[157] Doria-Rose NA, Joyce MG. 2015. Strategies to guide the antibody affinity maturation process. Curr Opin Virol 11:137–147. doi:10.1016/j.coviro.2015.04.002 25913818 PMC4456294

[158] Cao Y, Wang J, Jian F, Xiao T, Song W, Yisimayi A, Huang W, Li Q, Wang P, An R, et al.. 2022. Omicron escapes the majority of existing SARS-CoV-2 neutralizing antibodies. Nature 602:657–663. doi:10.1038/s41586-021-04385-3 35016194 PMC8866119

[159] Planas D, Saunders N, Maes P, Guivel-Benhassine F, Planchais C, Buchrieser J, Bolland W-H, Porrot F, Staropoli I, Lemoine F, et al.. 2022. Considerable escape of SARS-CoV-2 variant Omicron to antibody neutralization. Nature 602:671–675. doi:10.1038/s41586-021-04389-z 35016199

[160] Corbett KS, Edwards DK, Leist SR, Abiona OM, Boyoglu-Barnum S, Gillespie RA, Himansu S, Schäfer A, Ziwawo CT, DiPiazza AT, et al.. 2020. SARS-CoV-2 mRNA vaccine design enabled by prototype pathogen preparedness. Nature 586:567–571. doi:10.1038/s41586-020-2622-0 32756549 PMC7581537

[161] Wrapp D, Wang N, Corbett KS, Goldsmith JA, Hsieh C-L, Abiona O, Graham BS, McLellan JS. 2020. Cryo-EM structure of the 2019-nCoV spike in the prefusion conformation. Science 367:1260–1263. doi:10.1126/science.abb2507 32075877 PMC7164637

[162] Pallesen J, Wang N, Corbett KS, Wrapp D, Kirchdoerfer RN, Turner HL, Cottrell CA, Becker MM, Wang L, Shi W, Kong WP, Andres EL, Kettenbach AN, Denison MR, Chappell JD, Graham BS, Ward AB, McLellan JS. 2017. Immunogenicity and structures of a rationally designed prefusion MERS-CoV spike antigen. Proc Natl Acad Sci U S A 114:E7348–E7357. doi:10.1073/pnas.1707304114 28807998 PMC5584442

[163] Tanriover MD, Doğanay HL, Akova M, Güner HR, Azap A, Akhan S, Köse Ş, Erdinç FŞ, Akalın EH, Tabak ÖF, et al.. 2021. Efficacy and safety of an inactivated whole-virion SARS-CoV-2 vaccine (CoronaVac): interim results of a double-blind, randomised, placebo-controlled, phase 3 trial in Turkey. Lancet 398:213–222. doi:10.1016/S0140-6736(21)01429-X 34246358 PMC8266301

[164] Alter G, Yu J, Liu J, Chandrashekar A, Borducchi EN, Tostanoski LH, McMahan K, Jacob-Dolan C, Martinez DR, Chang A, et al.. 2021. Immunogenicity of Ad26.CoV2.S vaccine against SARS-CoV-2 variants in humans. Nature 596:268–272. doi:10.1038/s41586-021-03681-2 34107529 PMC8357629

[165] Chung AW, Kumar MP, Arnold KB, Yu WH, Schoen MK, Dunphy LJ, Suscovich TJ, Frahm N, Linde C, Mahan AE, Hoffner M, Streeck H, Ackerman ME, McElrath MJ, Schuitemaker H, Pau MG, Baden LR, Kim JH, Michael NL, Barouch DH, Lauffenburger DA, Alter G. 2015. Dissecting polyclonal vaccine-induced humoral immunity against HIV using systems serology. Cell 163:988–998. doi:10.1016/j.cell.2015.10.027 26544943 PMC5490491

[166] Miller J, Hachmann NP, Collier A-RY, Lasrado N, Mazurek CR, Patio RC, Powers O, Surve N, Theiler J, Korber B, Barouch DH. 2023. Substantial neutralization escape by SARS-CoV-2 Omicron variants BQ.1.1 and XBB.1. N Engl J Med 388:662–664. doi:10.1056/NEJMc2214314 36652339 PMC9878581

[167] Planas D, Bruel T, Staropoli I, Guivel-Benhassine F, Porrot F, Maes P, Grzelak L, Prot M, Mougari S, Planchais C, Puech J, Saliba M, Sahraoui R, Fémy F, Morel N, Dufloo J, Sanjuán R, Mouquet H, André E, Hocqueloux L, Simon-Loriere E, Veyer D, Prazuck T, Péré H, Schwartz O. 2023. Resistance of Omicron subvariants BA.2.75.2, BA.4.6, and BQ.1.1 to neutralizing antibodies. Nat Commun 14:824. doi:10.1038/s41467-023-36561-6 36788246 PMC9926440

[168] Wang Q, Iketani S, Li Z, Liu L, Guo Y, Huang Y, Bowen AD, Liu M, Wang M, Yu J, Valdez R, Lauring AS, Sheng Z, Wang HH, Gordon A, Liu L, Ho DD. 2023. Alarming antibody evasion properties of rising SARS-CoV-2 BQ and XBB subvariants. Cell 186:279–286. doi:10.1016/j.cell.2022.12.018 36580913 PMC9747694

[169] Viana R, Moyo S, Amoako DG, Tegally H, Scheepers C, Althaus CL, Anyaneji UJ, Bester PA, Boni MF, Chand M, et al.. 2022. Rapid epidemic expansion of the SARS-CoV-2 Omicron variant in Southern Africa. Nature 603:679–686. doi:10.1038/s41586-022-04411-y 35042229 PMC8942855

[170] Pajon R, Doria-Rose NA, Shen X, Schmidt SD, O’Dell S, McDanal C, Feng W, Tong J, Eaton A, Maglinao M, et al.. 2022. SARS-CoV-2 Omicron variant neutralization after mRNA-1273 booster vaccination. N Engl J Med 386:1088–1091. doi:10.1056/NEJMc2119912 35081298 PMC8809504

[171] Lauring AS, Tenforde MW, Chappell JD, Gaglani M, Ginde AA, McNeal T, Ghamande S, Douin DJ, Talbot HK, Casey JD, et al.. 2022. Clinical severity of, and effectiveness of mRNA vaccines against, covid-19 from omicron, delta, and alpha SARS-CoV-2 variants in the United States: prospective observational study. BMJ 376:e069761. doi:10.1136/bmj-2021-069761 35264324 PMC8905308

[172] Tegally H, Moir M, Everatt J, Giovanetti M, Scheepers C, Wilkinson E, Subramoney K, Makatini Z, Moyo S, Amoako DG, et al.. 2022. Emergence of SARS-CoV-2 Omicron lineages BA.4 and BA.5 in South Africa. Nat Med 28:1785–1790. doi:10.1038/s41591-022-01911-2 35760080 PMC9499863

[173] Hachmann NP, Miller J, Collier A-R, Ventura JD, Yu J, Rowe M, Bondzie EA, Powers O, Surve N, Hall K, Barouch DH. 2022. Neutralization escape by SARS-CoV-2 Omicron subvariants BA.2.12.1, BA.4, and BA.5. N Engl J Med 387:86–88. doi:10.1056/NEJMc2206576 35731894 PMC9258748

[174] McMahan K, Yu J, Mercado NB, Loos C, Tostanoski LH, Chandrashekar A, Liu J, Peter L, Atyeo C, Zhu A, et al.. 2021. Correlates of protection against SARS-CoV-2 in rhesus macaques. Nature 590:630–634. doi:10.1038/s41586-020-03041-6 33276369 PMC7906955

[175] Bartsch YC, Wang C, Zohar T, Fischinger S, Atyeo C, Burke JS, Kang J, Edlow AG, Fasano A, Baden LR, Nilles EJ, Woolley AE, Karlson EW, Hopke AR, Irimia D, Fischer ES, Ryan ET, Charles RC, Julg BD, Lauffenburger DA, Yonker LM, Alter G. 2021. Humoral signatures of protective and pathological SARS-CoV-2 infection in children. Nat Med 27:454–462. doi:10.1038/s41591-021-01263-3 33589825 PMC8315827

[176] Atyeo C, Fischinger S, Zohar T, Slein MD, Burke J, Loos C, McCulloch DJ, Newman KL, Wolf C, Yu J, Shuey K, Feldman J, Hauser BM, Caradonna T, Schmidt AG, Suscovich TJ, Linde C, Cai Y, Barouch D, Ryan ET, Charles RC, Lauffenburger D, Chu H, Alter G. 2020. Distinct early serological signatures track with SARS-CoV-2 survival. Immunity 53:524–532. doi:10.1016/j.immuni.2020.07.020 32783920 PMC7392190

